# Adverse Events Associated With Anti-IL-23 Agents: Clinical Evidence and Possible Mechanisms

**DOI:** 10.3389/fimmu.2021.670398

**Published:** 2021-06-11

**Authors:** Yi Ru, Xiaojie Ding, Ying Luo, Hongjin Li, Xiaoying Sun, Mi Zhou, Yaqiong Zhou, Le Kuai, Meng Xing, Liu Liu, Yue Luo, Jiankun Song, Jiale Chen, Bin Li, Xin Li

**Affiliations:** ^1^ Department of Dermatology, Yueyang Hospital of Integrated Traditional Chinese and Western Medicine, Shanghai University of Traditional Chinese Medicine, Shanghai, China; ^2^ Institute of Dermatology, Shanghai Academy of Traditional Chinese Medicine, Shanghai, China; ^3^ Shanghai Skin Disease Hospital, School of Medicine, Tongji University, Shanghai, China

**Keywords:** anti-IL-23, adverse events, meta-analysis, systematic review, biologics

## Abstract

**Background:**

Anti-interleukin (IL)-23 agents are widely used for autoimmune disease treatment; however, the safety and risks of specific symptoms have not been systematically assessed.

**Objectives:**

The aim of this study was to summarize the characteristics and mechanisms of occurrence of five immunological and non-immunological adverse events caused by different anti-IL-23 agents.

**Methods:**

The Cochrane Library, EMBASE, PubMed, and Web of Science databases were searched for eligible randomized clinical trials published from inception through May 1, 2020. Randomized clinical trials that reported at least one type of adverse event after treatment were included, regardless of sex, age, ethnicity, and diagnosis. Two investigators independently screened and extracted the characteristics of the studies, participants, drugs, and adverse event types. The Cochrane Handbook was used to assess the methodological quality of the included randomized clinical trials. Heterogeneity was assessed using the *I^2^* statistic. Meta-regression was applied to determine the sources of heterogeneity, and subgroup analysis was used to identify the factors contributing to adverse events.

**Results:**

Forty-eight studies were included in the meta-analysis, comprising 25,624 patients treated with anti-IL-23 agents. Serious immunological or non-immunological adverse events were rare. Anti-IL-12/23-p40 agents appeared to cause adverse events more easily than anti-IL-23-p19 agents. The incidence of cancer did not appear to be related to anti-IL-23 agent treatment, and long-term medication could lead to mental diseases. The prevention of complications should be carefully monitored when administered for over approximately 40 weeks to avoid further adverse reactions, and the incidence of infection was the highest among general immunological adverse events.

**Conclusions:**

The application of anti-IL-23 agents induced a series of immunological and non-immunological adverse events, but these agents tend to be well-tolerated with good safety profiles.

## Introduction

Autoimmune disorders represent a series of long-standing conditions with distinct appearances and characteristics. The mechanisms underlying central tolerance, peripheral tolerance, and adaptation ensure proper regulation of the immune system in healthy individuals to prevent autoimmunity (1). Current medical strategies for the treatment of autoimmune disorders mainly include nonsteroidal anti-inflammatory drugs, steroidal anti-inflammatory drugs, and disease-modifying anti-rheumatic drugs. However, these therapeutics are not effective in all patients, have undesirable adverse events (AEs), and fail to completely cure the diseases. Once symptoms appear, patients typically desire the resolution of pain rather than prevention of further onset. At the same time, biological agents (BAs) targeting cytokines, receptors, and signaling molecules that have been developed can overcome the limitations due to the multidrug resistance. In 1998, the Food and Drug Administration approved etanercept, a recombinant fusion protein of tumor necrosis factor receptor 2 with the Fc portion of human IgG1, as the first-generation BA for rheumatoid arthritis treatment. Since then, BAs have ushered in a new era in the treatment of autoimmune disorders. Consequently, the safety and tolerability of BAs in long-term and daily practice warrant more attention than ever.

BAs play a therapeutic role in blocking key inflammatory cytokines or cell-surface molecules (2). Their action mechanisms differ from those of chemical drugs and BAs are not digested in the gastrointestinal tract (3) Most BAs are naturally occurring proteins or humanized antibodies that can neutralize natural proteins, which can result in AEs. In contrast to those elicited by chemicals, AEs caused by BAs mainly depend on the chemistry, mode of action, metabolism, and immunogenicity (4). To distinguish the AEs caused by BAs from other adverse effects, AEs are classified into five types using the Greek alphabet (type α to ***ϵ***) (3) (**Table S1**). Type α AEs occur after the abundant release of inflammatory factors with complicated and changeable symptoms. Type β AEs are immune-mediated and more serious than type α AEs. Type γ and δ AEs involve short-term and long-term toxicities, respectively, linked to the chemical structure of BAs and their metabolism. Type ***ϵ*** AEs occur during drug withdrawal, particularly when the drug is suddenly stopped.

Interleukin (IL)-23 affects inflammatory cells and relies on the ability of cytokines to indirectly counteract regulatory mechanisms. The anti-IL-23 agents has good safety and clinical curative effect (5). Ustekinumab was recognized as the most widely used anti-IL-12/23-p40 agent, which was approved in 2009 for the treatment of psoriasis (Pso) with advantages of few drug injections, high remission rates, and long-term sustainment. Although blocking the IL-23 immune axis is sufficient to treat many autoimmune disorders, the risk of serious infections and various AEs is a concern (3, 4). A phase III trial demonstrated that briakinumab, a fully human monoclonal antibody directed against IL-12/23-p40 for psoriasis treatment, caused serious complications and AEs, and the drug developer withdrew its approval application submitted to the Food and Drug Administration and European Agency for the Evaluation of Medicinal Products in 2011 (6, 7). This incident resulted in controversy and additional scrutiny of anti-IL-23 agents. In a review of four commercial BAs, secukinumab (an IL-17 inhibitor) and ustekinumab were suggested to achieve better effects (8). Another study also proposed that the probability of AEs with anti-IL-23 agents is lower than that with anti-IL-17 agents (9). However, summaries of the safety of anti-IL-23 agents are not available; thus, further analysis is necessary.

The aim of this systematic review and meta-analysis was to assess the incidence and characteristics of AEs caused by all anti-IL-23 agents currently available in the market (**Table 1**), and to provide a comprehensive synopsis based on existing evidence, of the efficacy and safety of anti-IL-23 agents, which will help to identify future research priorities.

**Table 1:** Summary of Biological Agents Targeting IL-23.

Name	Trade name	subunit	Constituent	Indication	R & D stage	Identifier
Ustekinumab	Stelara	IL-23/12-p40	a fully human IgG1κ monoclonal antibody	Psoriasis	Phase IV completed	NCT01059773
				Crohn’s disease	Phase IV ongoing	NCT03885713
				Ankylosing spondylitis	Phase III terminated	NCT01330901
				Rheumatoid arthritis	Phase II completed	NCT01645280
				Psoriatic arthritis	Phase III completed	NCT01077362
				Multiple sclerosis	Phase II completed	NCT00207727
				Graft-versus-host disease	Phase II completed	NCT01713400
				Atopic dermatitis	Phase II completed	NCT01806662
				Giant cell arteritis	Phase II terminated	NCT02955147
				Type I diabetes mellitus	Phase II completed	NCT02204397
				Systemic lupus erythematosus	Phase III ongoing	NCT04060888
				Hidradenitis suppurativa	Phase II completed	NCT01704534
				Sarcoidosis	Phase II completed	NCT00955279
				Primary biliary cirrhosis	Phase II completed	NCT01389973
Briakinumab	–	IL-23/12-p40	a fully human IgG1λ monoclonal antibody	Crohn’s disease	Phase II terminated	NCT00562887
				Psoriasis	Phase III completed	NCT00626002
				Multiple sclerosis	Phase II completed	NCT00086671
Guselkumab	Tremfya	IL-23-p19	a fully human IgG1λ monoclonal antibody	Psoriasis	Phase IV completed	NCT03573323
				Rheumatoid arthritis	Phase II completed	NCT01645280
				Palmoplantar pustulosis	Phase III completed	NCT02641730
Tildrakizumab	Ilumya	IL-23-p19	humanized IgG1κ monoclonal antibody	Psoriasis	Phase IV ongoing	NCT04339595
				Psoriatic arthritis	Phase III ongoing	NCT04314531
Risankizumab	Skyrizi	IL-23-p19	humanized IgG1 monoclonal antibody	Ankylosing spondylitis	Phase II completed	NCT02047110
				Crohn’s disease	Phase III ongoing	NCT03105128
				Psoriasis	Phase IV ongoing	NCT04102007
				Psoriatic arthritis	Phase III ongoing	NCT03675308
				Asthma	Phase II completed	NCT02443298
				Ankylosing spondylitis	Phase II completed	NCT02047110
Brazikumab	–	IL-23-p19	humanized IgG2 monoclonal antibody	Crohn’s disease	Phase III ongoing	NCT03961815
				Psoriasis	Phase I completed	NCT01094093
				Ulcerative colitis	Phase II ongoing	NCT04277546
Mirikizumab	–	IL-23-p19	humanized IgG4 monoclonal antibody	Psoriasis	Phase III ongoing	NCT03535194
				Ulcerative colitis	Phase III ongoing	NCT03519945
				Crohn’s disease	Phase III ongoing	NCT03926130

R & D stage, Research and development stage; Ig, immunoglobulin.

*Clinical trial identifiers are provided for reference; please see the ClinicalTrials.gov website for further details. Data are accurate as of June 2020.

## Methods

This systematic review was performed following the Cochrane Handbook for Systematic Reviews of Interventions (9), and is presented according to the Preferred Reporting Items for Systematic Reviews and Meta-Analyses (PRISMA) guidelines.

### Selection Criteria

The inclusion and exclusion criteria were determined before initiating the study. The included studies were limited to randomized clinical trials (RCTs) that reported at least one type of AE after anti-IL-23 agent treatment, regardless of sex, age, ethnicity, and diagnosis. The exclusion criteria were as follows: (i) no anti-IL-23 agents included; (ii) no reported AEs; (iii) meeting abstracts, cell or animal studies, reviews, systematic reviews, and meta-analyses; and (iv) no full-text studies.

### Outcomes

The primary outcomes considered were the incidence and grade of AEs according to the Common Terminology Criteria for Adverse Events v5.0 (updated November 2017) of the Department of Health and Human Services. AEs of grades ≥ 3 were considered severe. Heterogeneity and AE incidence were evaluated using meta-regression and subgroup analyses. The primary outcomes included general information of the RCTs, and the secondary outcomes were measured according to the five AE types.

### Selection of Studies and Data Extraction

The Cochrane Library, Excerpta Medica database (EMBASE), PubMed, and Web of Science databases were searched for studies published from inception through May 1, 2020, without language restrictions. The details of the clinical trials were searched at ClinicalTrials.gov until June 1, 2020. The search terms were grouped into three blocks (10).Character included “safety”, “side effects”, “adverse reactions” or “adverse events”. Clinical condition included “ustekinumab or stelara or CNTO 1275” or “briakinumab or ABT874” or “guselkumab or tremfya or CNTO1959” or “tildrakizumab or ilumya or SCH900222” or “risankizumab or skyrizi or ABBV 066” or “brazikumab or MEDI 2070” or “miriklzumab”. Trial design included clinical trial, random, and control. Vocabulary and syntax were adapted to be appropriate for each database. In total, 4,285 potential studies were identified from the electronic databases, and the detailed steps for study selection are shown in **Figure S1**.

### Quality Assessments

The quality of the studies was assessed according to the Cochrane Handbook (9), including: random sequence generation, allocation concealment, blinding of participants and personnel, blinding of the outcome assessment, incomplete outcome data, selective reporting, and other biases. The terms “low”, “unclear”, “high”, and “n/a” referred to low, uncertain, high risks of bias, and not applicable, respectively. The results were cross-checked by two investigators (YR, XJD) and disagreements were settled under discussion. Potential publication bias was detected by visual inspection of a funnel plot and formal testing with Egger’s test. Meta-regression was performed to explore the sources of study heterogeneity.

### Statistical Analysis

We estimated the incidence of AEs associated with anti-IL-23 agent treatment. Heterogeneity between studies was assessed using the Q test and *I^2^* statistic. The random-effects model was used to calculate the average statistics of the weighted combination of multiple research statistics. All analyses were performed using R software (version 3.8.6 [2018–3–15]) with the package Meta and Metafor function, and results were assessed at a significance level of *P* < 0.05.

## Results

In total, 48 studies reported at least one type of AE caused by anti-IL-23 agents. The articles were retrieved from the Cochrane Library, EMBASE, PubMed, and Web of Science databases. A flowchart of the search process is presented in **Figure S1**. All included studies were RCTs. When a study had more than one dosage cohort, each cohort was used as an independent study for analysis.

### Quality and Characteristics of the Studies

Visual inspection of the funnel plot showed no evident asymmetry, indicating that the pooled results were not influenced by publication bias (**Figure S2**). Egger’s test showed *P* = 0.3226, indicating no evidence of publication bias (**Figure S3**). The overall AE incidence revealed high between-study heterogeneity with *I^2^* = 98%; the odds ratio (OR) was 64.76% with a 95% confidence interval (CI) of 60.75–68.78 (**Figure S4**). The quality of most RCTs was high, according to the Cochrane quality assessment criteria (**Table S2**). The study population characteristics are shown in **Table 2**. To explore the potential sources of heterogeneity, meta-regression analysis was performed for the endpoints of AEs in different groups (**Table 3**).

**Table 2:** Study population characteristics: the adverse events caused by anti-IL-23 agents.

Study	Region	Design(Phase)	Diagnosis	Drug/Target	Total number of patients (M/F)	Dose(mg/kg)	mode of administration	CTCAE(Grade)
Krueger et al. (11)	U.S.A., Utah	II	Ps	Ustekinumab/p40	320 (222/98)	0.75; 1.5	i.h.	3
Papp et al. (12)	Canada, Ontario	III	Ps	Ustekinumab/p40	1230 (840/390)	0.75; 1.5	i.h.	3
Leonardi et al. (13)	U.S.A., Skokie	III	Ps	Ustekinumab/p40	766 (531/235)	0.75; 1.5	i.h.	3
Griffiths et al. (14)	U.K., Manchester	III	Ps	Ustekinumab/p40	903 (613/290)	0.75; 1.5	NA	3
Igarashi et al. (15)	Japan, Tokyo	II/III	Ps	Ustekinumab/p40	158 (126/32)	0.75; 1.5	i.h.	3
Tsai et al. (16)	Taiwan; Korea	III	Ps	Ustekinumab/p40	121 (103/18)	0.75	i.h.	3
Gordon et al. (6)	U.S.A., Skokie	III	Ps	Briakinumab/p40	Induction: 1465 (1009/456) Maintenance:745 (507/238)	3.33; 1.67	NA	4
Kimball et al. (17)	U.S.A., Chicago	III	Ps	Ustekinumab/p40	766 (531M235)	0.75; 1.5	NA	3
Sandborn et al. (18)	U.S.A., California	IIa	CD	Ustekinumab/p40	526 (217/309)	1; 1.5; 3; 6	i.v./i.h.	3
Kimball et al. (19)	U.S.A., Boston	III	Ps	Ustekinumab/p40	753 (523M230)	0.75; 1.5	i.h.	3
McInnes et al. (20)	U.K., Glasgow	III	PsA	Ustekinumab/p40	615 (330/285)	0.75; 1.5	i.h.	3
Zhu et al. (21)	U.S.A. North Dakota	I	Healthy	Ustekinumab/p40	55 (55/0)	1.5;0.75	i.h.	2
Langley et al. (22)	Canada, Nova Scotia	III	Ps	Ustekinumab/p40	1212 (828/384)	0.75; 1.5	NA	3
Ritchlin et al. (23)	U.S.A., Rochester	III	PsA	Ustekinumab/p40	312 (148/164)	0.75; 1.5	i.h.	3
Sofen et al. (24)	U.S.A., New York	I	Ps	Guselkumab/p19	24 (15/9)	0.17; 0.5; 1.67; 5	i.h.	2
Gordon et al. (25)	U.S.A., Chicago	II	Ps	Guselkumab/p19	293 (207/86)	0.08; 0.25; 0.83; 1.67; 3.33	i.h.	4
Kavanaugh et al. (26)	U.S.A., California	III	PsA	Ustekinumab/p40	615 (330/285)	0.75; 1.5	NA	3
Kopp et al. (27)	Austria, Vienna	I	Ps	Tildrakizumab/p19	77 (61/16)	0.05; 0.1; 0.5; 3; 10	i.v.	3
Landells et al. (28)	Canada,Newfoundland	III	Ps	Ustekinumab/p40	110 (54/56)	0.75; 1.5	i.h.	3
Papp et al. (29)	Canada, Waterloo	IIb	Ps	Tildrakizumab/p19	355 (270/85)	0.08; 0.42; 1.67; 3.33	i.h.	4
Thaçi et al. (30)	Germany, Schleswig- Holstein	III	Ps	Ustekinumab/p40	676 (252/424)	0.75; 1.5	i.h	3
Blauvelt et al. (31)	U.S.A., Oregon	IIIb	Ps	Ustekinumab/p40	676 (252/424)	0.75; 1.5	i.h	4
Blauvelt et al. (32)	U.S.A., Oregon	III	Ps	Guselkumab/p19	837 (608/129)	1.67	NA	3
Feagan et al. (33)	U.K., London	IIb	CD	Ustekinumab/p40	UNIT-1: 741 (317/424) UNIT-2: 628 (293/335) IM-UNIT-I: 397 (173/224)	2.17; 6; 1.5	i.v.	3
Zhuang et al. (34)	U.S.A., Pennsylvania	I	Ps	Guselkumab/p19	Part 1: 47 (45/2) Part 2: 24 (15/9)	0.03; 0.1; 0.17; 0.3; 0.5; 1; 1.67; 3; 5; 10	i.v.	2
Blauvelt et al. (35)	U.S.A., Portland	IIIb	Ps	Ustekinumab/p40	378 (236/142)	NA	i.h.	3
Papp et a. (36)	Canada, Waterloo	II	Ps	Risankizumab/p19 Ustekinumab/p40	166 (109/57)	0.3; 1.5; 3; 0.75	i.h.	3
Reich et al. (37)	Germany, Hamburg	IIIb	Ps	Ustekinumab/p40	302 (202/100)	0.75; 1.5	i.h.	3
Reich et al. (38)	Germany, Hamburg	III	Ps	Guselkumab/p19	992 (692/300)	1.67	i.h.	3
Reich et al. (39)	Germany, Hamburg	III	Ps	Tildrakizumab/p19	reSURFACE 1: 772 (533/239) reSURFACE 2: 1090 (779/311)	1.67; 3.33	i.h.	4
Saeki et al. (40)	Japan, Tokyo	II	AD	Ustekinumab/p40	79 (55/24)	0.75; 1.5	i.h.	2
Deodhar et al. (41)	U.S.A., Oregon	II	PsA	Guselkumab/p19	149 (76/73)	1.67	i.h.	3
Nemoto et al. (42)	Japan, Tokyo	I	Ps	Guselkumab/p19	24 (18/6)	0.17; 0.5; 1.67; 5	i.h.	2
Ohtsuki et al. (43)	Japan, Tokyo	III	Ps	Guselkumab/p19	192 (145/47)	0.83; 1.67	i.h.	3
Paul et al. (44)	France, Toulouse	IIIb	Ps	Ustekinumab/p40	302 (202/100)	0.75; 1.5	i.h.	3
Terui et al. (45)	Japan, Tokyo	II	PP	Guselkumab/p19	49 (14/35)	3.33	i.h.	3
Ferris et al. (46)	U.S.A., Pittsburgh	III	Ps	Guselkumab/p19	78 (53/25)	1.67	i.h.	3
Lee et al. (47)	Korea, Seoul	III	Ps	Ustekinumab/p40	62 (49/13)	0.75; 1.5	i.h.	3
Ohtsuki et al. (48)	Japan, Tokyo	II/III	Ps	Risankizumab/p19	171 (143/28)	1.25; 2.5	NA	3
Reich et al. (49)	Germany, Berlin	II	Ps	Mirikizumab/p19	205 (152/53)	0.5; 1.67; 5	i.h.	3
Sandborn et al. (50)	U.S.A., California	II	UC	Mirikizumab/p19	249 (149/100)	0.83; 3.33; 10	i.v./i.h.	3
Sands et al. (51)	U.S.A., New York	III	UC	Ustekinumab/p40	Induction: 961 (582/379) Maintenance: 523 (297/226)	2.17; 6; 1.5	i.v.	4
Terui et al. (52)	Japan, Tokyo	III	PP	Guselkumab/p19	159 (30/129)	1.67; 3.33	i.h.	3
Blauvelt et al. (53)	U.S.A., Oregon	IV	Ps	Guselkumab/p19	1027 (652/375)	1.67	i.h.	3
Gelfand et al. (54)	U.S.A., Pennsylvania	IV	Ps	Ustekinumab/p40	43 (30/13)	0.75; 1.5	i.h.	2
Reich et al. (55)	Germany, Hamburg	III	Ps	Tildrakizumab/p19	772 (533/239)	1.67; 3.33	i.h.	4
Thaçi et al. (56)	Germany, Lubeck	IIIb	Ps	Guselkumab/p19	119 (82/37)	1.67	i.h.	3
Blauvelt et al. (57)	U.S.A., Oregon	III	Ps	Risankizumab/p19	Part A: 507 (356/151) Part B: 336 (239/97)	2.5	i.h.	4

AD, atopic dermatitis; CD, Crohn’s disease; PP, palmoplantar pustulosis; Ps, psoriasis; PsA, psoriatic arthritis; UC, ulcerative colitis; CTCAE, Common Terminology Criteria for Adverse Events v5.0; U.S.A., United States of America; U.K., United Kingdom; NA, non-available; M/F, Male/Female; i.v., intravenous injection; i.h., hypodermic injection.

**Table 3:** Potential prespecified sources of heterogeneity explored among the studies reporting AEs associated with anti-IL-23 agents.

Prespecified Source of Heterogeneity	Number of studies	Meta-regression coefficient with 95% confidence interval	Meta-regression *P*-value for Heterogeneity
**Region**			0.037
U.S.A	20	1.22 [0.99,1.51]	
Europe	11	1.17 [0.93,1.47]	
Asia	9	1.31 [1.03,1.67]	
Other	5	0.82 [0.67,0.99]	
**Action Subunits**			0.226
p19	22	0.93 [0.82,1.05]	
p40	27	1.08 [0.95,1.23]	
**Clinical Phase Design**			0.133
Phase I clinical	5	1.00 [0.69,1.44]	
Phase II clinical	11	0.92 [0.67,1.24]	
Phase II/III clinical	2	1.10 [0.83,1.45]	
Phase III clinical	26	0.90 [0.68,1.20]	
Phase IV clinical	1	0.82 [0.45,1.50]	
**Sample Size**			0.004
≤ 500	27	1.12 [0.99,1.27]	
> 500	18	0.89 [0.79,1.01]	
**Administration**			0.983
i.v.	7	1.10 [0.91,0.87]	
i.h.	33	0.91 [0.75.1.11]	
NA	7	0.89 [0.71.1.13]	
**Diseases**			0.015
Atopic dermatitis	1	1.38 [0.60,3.20]	
Crohn’s disease	2	1.49 [0.77,2.92]	
Palmoplantar pustulosis	2	1.54 [0.74,3.19]	
Psoriasis	33	1.31 [0.71,2.43]	
Psoriasis arthritis	4	1.20 [0.63,2.29]	
Ulcerative colitis	2	1.28 [0.66,2.50]	
Health	1	0.76 [0.42,1.39]	
**Courses of Medication**			0.690
≤ half a year	15	1.04 [0.89,1.21]	
> half a year	30	0.96 [0.55,0.73]	
**Dose Adjustment**			0.258
Yes	30	0.91 [0.79,1.05]	
No	15	1.10 [0.95,1.27]	
**Study Quality**			0.773
Low risk	34	0.99 [0.84,1.15]	
High risk	1	1.35 [0.78,2.35]	
Uncertain risk	10	1.01 [0.86,1.82]	

AEs, Adverse Events; CI, Confidence Interval; i.v., intravenous injection; i.h., hypodermic injection; NA, non-available.

### Effect of Duration of Anti-IL-23 Treatment

**Figure 1:**
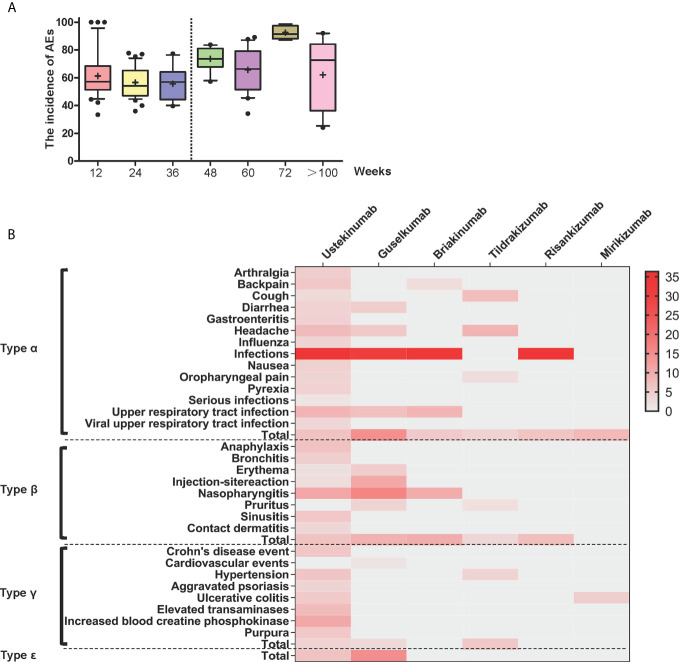
**(A)** Box diagram for subgroup analysis. Box diagram for the incidence of changes in adverse events with different medication courses. **(B)** Heat map of different types of symptoms caused by different drugs. Red areas indicate greater relative probability of occurrence and lighter colored areas indicate a slight or null relative probability of occurrence. All data included in the heat map are statistically significant (*P* < 0.05).

Forty-five studies proposed a clear definition of medication courses. We sorted the AE incidence and found a high degree of heterogeneity among different treatment courses (**Figure S6.3**). To describe the characteristics of medication courses in-depth, each interval at 12 weeks was marked, and a line chart was created with an approximately equal number of courses (**Figure 1A**). The AE incidence was lower when the medication period lasted within three-quarters of a year (approximately 42 weeks) (**Figure 1A**).

### Targeted Subunits and Commercial Drugs

Further subgroup analysis showed that the AE occurrence of anti-IL-23/IL-12-p40 agents was 65.23% (95% CI 61.74–68.57, *I^2^* = 95%), which was higher than that of anti-IL-23-p19 agents of 58.71% (95% CI 55.40–61.94, *I^2^* = 91%) (**Table S3** and **Figure S6.5.1**). The evident differences in AE incidence were as follows: 71.56% (95% CI 66.68–75.98, *I^2^* = 96%) for ustekinumab, 67.07% (95% CI 55.53–76.87, *I^2^* = 88%) for briakinumab, 65.69% (95% CI 54.44–75.42, *I^2^* = 96%) for risankizumab, 59.13% (95% CI 53.77–64.28, *I^2^* = 79%) for guselkumab, and 51.19% (95% CI 45.80–56.56, *I^2^* = 89%) for tildrakizumab (**Table S3** and **Figure S6.5.2**). However, the results were also affected by the actual clinical usage.

### Incidence of the Five Reaction Types

The pooled OR from the random-effects model for the five AE types associated with anti-IL-23 agents was analyzed. The incidence of type ***ϵ*** AEs was 13.3% (95% CI 6.23–26.17, *I^2^* = 77%), which was higher than that of type α AEs with an incidence of 7.14% (95% CI 6.41–7.93, *I^2^* = 97%), type β AEs at 6.57% (95% CI 5.81–7.41, *I^2^* = 92%), and type γ AEs at 3.94% (95% CI 3.33–4.65, *I^2^* = 80%). However, there was no significant effect on the incidence of type δ AEs.

#### Type α AEs

The anti-IL-23-p19 agents tended to most strongly increase the risk for type α AEs among the five types. The most frequent type of α AE during treatment was infections with an incidence of 36.35%; serious infections showed an incidence of <1.5% (**Table S3** and **Figure S7.1.1**). The heat map shows darker areas with a high probability of incidence and lighter areas with a low probability of occurrence (**Figure 1B**). Notably, other common signs of type α reactions included upper respiratory tract infection (8.53%), headache (7.12%), and viral upper respiratory tract infection (6.73%). The other frequent type of α AEs included arthralgia, backache, cough, diarrhea, gastroenteritis, influenza, nausea, oropharyngeal pain, and pyrexia (**Table S3** and **Figure S7.1.1**).

#### Type β AEs

Anti-IL-23-p19 agents were more likely to induce type β AEs. The most common symptom was nasopharyngitis, with an incidence of 12.21%. Other AEs with a higher probability of occurrence included anaphylaxis (5.00%), sinusitis (4.57%), and pruritus (4.40%). Other common type β AEs were bronchitis, erythema, injection-site reaction, and neutropenia (**Table S3** and **Figure S7.2.1**).

#### Type γ AEs

Compared with the anti-IL-23-p19 agents, the anti-IL-12/23-p40 agents appeared to more frequently lead to type γ AEs. The most common type of γ AE symptom was increased transaminases (approximately 7.43%). Prolonged application of anti-IL-23 agents aggravated the original diseases, such as increasing the severity of Crohn’s disease (5.96%), hypertension (5.50%), ulcerative colitis (5.28%), Pso (3.92%), and cardiovascular events (0.76%) (**Table S4** and **Figure S7.3.1**).

#### Type ϵ AEs

Anti-IL-23-p19 agents increased the risk of type ***ϵ*** AEs more than anti-IL-12/23-p40 agents. The most frequent symptom was depression (0.75%; **Table S3** and **Figure S7.4.1**).

## Discussion

Biological therapy has evolved, owing to improved integration of knowledge of the interactions between the immune system and related cytokines, which could affect the entire pathologic disease process. Anti-IL-23 agents displayed broad range of antagonistic activities because IL-23 is a critical upstream regulator (58). IL-23 acts early in the disease inflammatory cascade, which activates downstream effectors to maintain the T_H_17 cell phenotype (59). Moreover, IL-12 is considered to be proatherogenic and the inhibition of IL-12/23-p40 should confer cardioprotection (60). A previous study (n = 2,447) (5) indicated that anti-IL-23 agents appear to be safer and more effective in clinical application, but the drug-specific safety information has not been explored systematically. This is the first report to classify and review the AEs caused by anti-IL-23 agents, and to provide a statistical outline.

**Figure 2:**
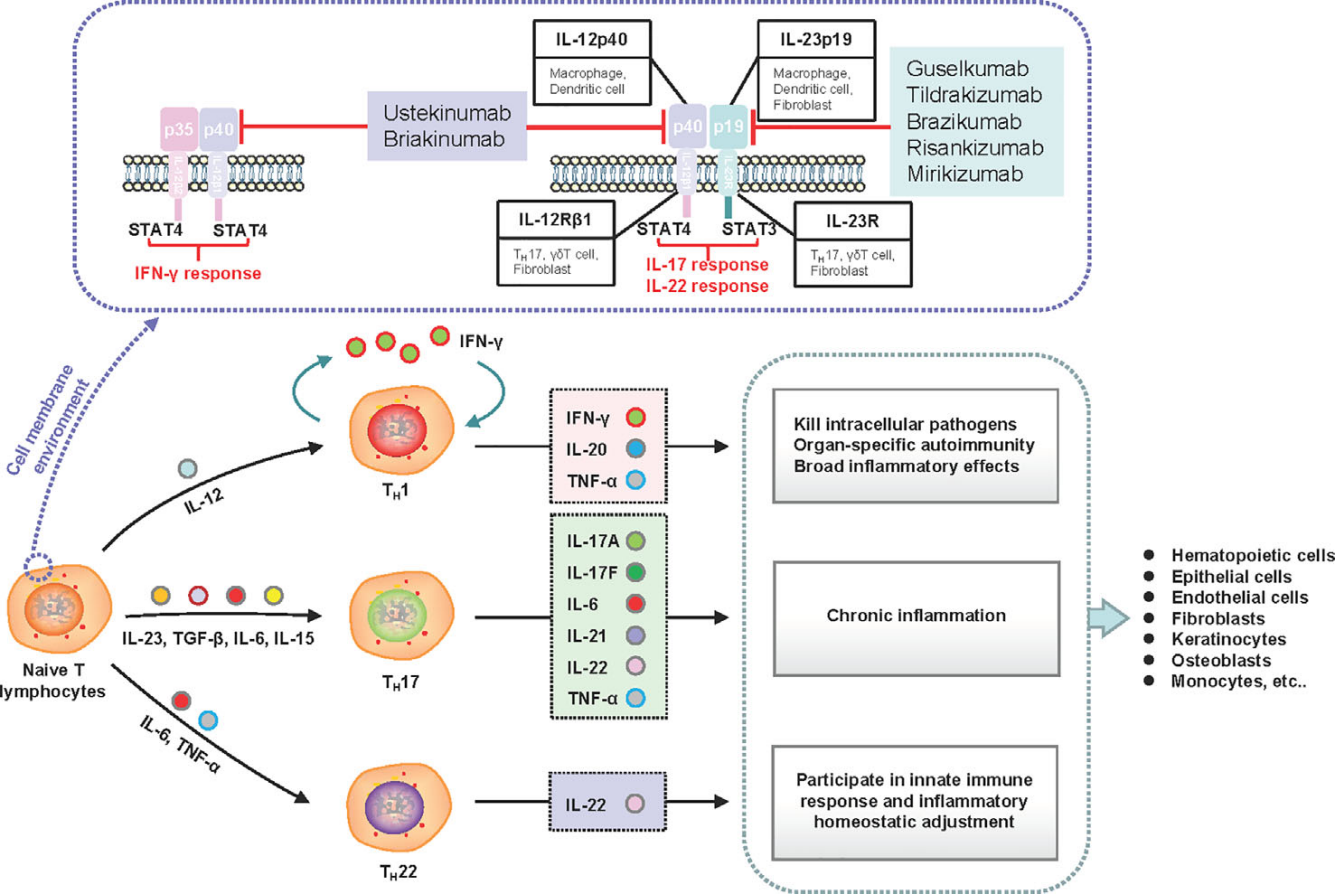
Schematic of the induction and effects of IL-23 subunits and their corresponding receptors and signaling molecules. IL-23 is an essential factor required for the expansion of naive CD4^+^ T lymphocyte populations. The functional IL-23 receptors include IL-12Rβ1 and IL-23R. The downstream signaling molecule of IL-23R is STAT3, which drives T_H_17 and T_H_22 responses. The downstream signaling molecule of IL-12Rβ1 is STAT4, which drives the T_H_1 response. Under stimulation with different cell factor combinations, naive CD4^+^ cells differentiate into T helper (T_H_1, T_H_17, and T_H_22) cells to produce corresponding inflammatory factors and execute their functional activities. Inhibiting the upstream subunits (IL-12Rβ1 or IL-23R) can govern both upstream and downstream processes in the cascade to improve clinical symptoms. IFN, interferon; IL, interleukin; STAT, signal transducer and activator of transcription; T_H_, T helper; TGF, transforming growth factor; TNF, tumor necrosis factor.

IL-23 is secreted by several immune cells in response to microbial products and inflammatory cytokines, which essentially bridge the innate and adaptive immune responses and drive early local immunity (61, 62). IL-23 is structurally composed of the unique IL-23-p19 subunit linked to the common p40 subunit that is shared with IL-12. Moreover, IL-23 and IL-12 are responsible for driving the differentiation of naïve T helper (T_H_) cells to T_H_17 and T_H_1 cells, respectively (63). T_H_17 cells secrete several proinflammatory cytokines, stimulate the proliferation of keratinocytes, and activate downstream inflammatory signal transduction (64). T_H_1 cells produce inflammatory cytokines and drive the expansion of inflammation. The inhibition of IL-23 blocks the cascade of both immune and inflammatory reactions (**Figure 2**). Primary subgroup analysis showed significant differences in the AE incidence of briakinumab, ustekinumab, guselkumab, risankizumab, and tildrakizumab (**Table S3** and **Figure S6.5.2**). Anti-IL-12/23-p40 agents (briakinumab and ustekinumab) were more likely to cause AEs with an incidence of 65.23% (95% CI 61.74–68.57, *I^2^* = 95%) (**Table S3** and **Figure S6.5.2**) than anti-IL-23-p19 treatment, which may be related to its structural features (65). Therefore, a higher rate of malignancy with IL-12/23-p40 blockade was confirmed (66); IL-12 can promote the infiltration of cytotoxic T cells and IL-23 can promote inflammatory responses (66). While our results showed that anti-IL-23 treatment was rarely associated with an increased cancer risk (**Figure S3**), similar those of a recent study (67). In summary, the AEs caused by anti-IL-23 agents were mainly due to their immunological effects and broad range of biological effects.

AEs induced by BAs always require specific knowledge, as they are different from the common AEs elicited by chemicals or drugs (**Table S1**). The most common AE identified in the included studies was type α with an incidence of 7.14% (95% CI 6.41–7.93, *I^2^* = 97%), and the highest risk was for type ***ϵ*** AEs (13.30%, 95% CI 6.23–26.17, *I^2^* = 89%) (**Table S3** and **Figure S7**). Type ***ϵ*** is the only non-immunological AE form, which may be associated with long-term medication. Previous studies (68, 69) have shown that excessive release of pathological inflammatory mediators may lead to a high incidence of anxiety and depression; moreover, the application of BAs induces psychologically relevant AEs. The most common type ***ϵ*** AE was psychiatric disorder with an incidence of 0.75% (**Table S3** and **Figure S7.5.1**). This indicates that the treat-and-extend regimens over a longer period may lead to mental problems, which may also relate to the chronic duration. In summary, clinician should pay attention to communication and psychological guidance to patients.

The curative effect is often comparable with decreasing tolerance to AEs. A drug concentration-response relationship affects the clinical efficacy, indicating that a higher BA drug dose may achieve better and longer efficacy duration in the first few weeks (70). However, no significant dose-dependent efficacy of anti-IL-23 agents was observed during long-term treatment (71). Regardless of whether drug dose affects the curative effect, dose adjustment showed no significant effect on AEs in our analysis (**Table 3**). Dose tapering does not lead to persistent flares or safety issues (72); however, variation in medication courses may induce heterogeneity. According to the subgroup analysis, the incidence of AEs stabilized when the medication period was maintained within 40 weeks (**Figure 1A**). A previous study (11) showed that long-term BA treatment may even decrease the survival rate due to serious AEs and ineffective treatments. This suggests that anti-IL-23 treatment is relatively safe in the first three quarters of medication use, and an intervention such as combined treatment for potential complications should be considered within the first 40 weeks.

Currently, with the increasing popularity of BA treatment, the development of new drugs is actively taking place. These drugs are beneficial for treating symptoms, and assessing the applicability of patients for potential AEs is an essential step; however, the assessment method remains unclear, and there is currently no consensus on the most appropriate treatment duration of BAs for different diseases. In addition, BAs cannot prevent the entire disease mechanism, which has raised the question as to whether BAs can change the natural course of the disease. Moreover, BAs are relatively expensive and therefore increase healthcare costs, which could be resolved in the near future with the approaching expiration of patents. Finally, no study has discussed the risk of biologics solely in specific subpopulations (such as pregnant women, children, and the elderly), which should be the subject of future research.

This study has several strengths. First, several authors reviewed all available studies independently for data retrieval and analysis to reduce information bias or missing data. Second, the included studies were RCTs of generally high quality, which minimized the selection bias associated with differences between researchers and medical settings to some extent. Third, long-term follow-up studies were included with significant implications that were found by collating rare or non-immune AEs caused by anti-IL-23 agents. Fourth, no industry was involved in this work. However, there were also some limitations to this study. The different treatment strategies had different time points used as safety measures, which prevented inclusion of placebo control data for analysis. To resolve the ambiguity of AEs caused by BAs, we prolonged the observation periods and used five defined types to screen for AEs caused by anti-IL-23 agents. In addition, we used type ***ϵ*** analysis to detect long-term and rare AEs, but this was not an in-depth exploration. Further studies should focus on the differences in AEs associated with autoimmune disorders over long-term treatment, which would certainly help to tackle remaining questions in this field.

## Data Availability Statement

The original contributions presented in the study are included in the article/**Supplementary Material**. Further inquiries can be directed to the corresponding authors.

## Author Contributions

Conceptualization: YR, XD, and BL. Data curation: XD and YiL. Formal analysis: LK, XS, and LL. Funding acquisition: XL and BL. Investigation: YR and YZ. Methodology: YR, XD, and YiL. Project Administration: HL and YuL. Resources: JS and MZ. Software: MX and LL. Supervision: BL and XL. Validation: LL and JC. Visualization: XL and BL. Writing – Original Draft Preparation: YR and XD. Writing – Review & Editing: XL and BL. All authors contributed to the article and approved the submitted version.

## Funding

This work was supported by the National Nature Science Foundation of China [grant numbers: 82074427,81874470, 81973860], National Key Research and Development Program of China [grant number: 2018YFC1705305], Science and Technology Commission of Shanghai Municipality [grant number: 19ZR1458700], the Shanghai Pujiang Talent Plan (No. 2020PJD067), Shanghai Development Office of TCM [No. ZY(2018-2020)-FWTX-1008], and Shanghai Municipal Key Clinical Specialty (No. shslczdzk05001).

## Conflict of Interest

The authors declare that the research was conducted in the absence of any commercial or financial relationships that could be construed as a potential conflict of interest.

## Supplementary Material

Supplementary Figure 1 Flow diagram of the literature search and selection.

Supplementary Figure 2 Funnel plot

Supplementary Figure 3 Egger’s test

Supplementary Figure 4 Incidence of total adverse events with different anti-IL-23 agents in all patients.

Supplementary Figure 5 Incidence of cancers with different anti-IL-23 agents in all patients.

Supplementary Figure 6.1 Incidence of adverse events in different regions

Supplementary Figure 6.2 Incidence of adverse events with different. diagnoses in all patients

Supplementary Figure 6.3 Incidence of adverse events with different courses of medication in all patients.

Supplementary Figure 6.4.1 Incidence of adverse events with different therapeutic doses in all patients.

Supplementary Figure 6.4.2 Incidence of adverse events with different frequency of application in all patients.

Supplementary Figure 6.4.3 Incidence of adverse events in the induction and maintenance periods in all patients.

Supplementary Figure 6.5.1 Incidence of adverse events with different targeted subunits in all patients.

Supplementary Figure 6.5.2 Incidence of adverse events with different anti-IL-23 agents in all patients.

Supplementary Figure 7 Incidence of adverse events of all the symptoms using different anti-IL-23 agents.

Supplementary Figure 7.1.1 Incidence of type α adverse events symptoms using different anti-IL-23 agents in all patients.

Supplementary Figure 7.1.2 Incidence of type α adverse events using different targeted subunits in all patients.

Supplementary Figure 7.1.3 Incidence of type α adverse events using different anti-IL-23 agents in all patients.

Supplementary Figure 7.2.1 Incidence of type β adverse events symptoms using different anti-IL-23 agents in all patients.

Supplementary Figure 7.2.2 Incidence of type β adverse events using different targeted subunits in all patients.

Supplementary Figure 7.2.3 Incidence of type β adverse events using different anti-IL-23 agents in all patients.

Supplementary Figure 7.3.1 Incidence of type γ adverse events symptoms using different anti-IL-23 agents in all patients.

Supplementary Figure 7.3.2 Incidence of type γ adverse events using different targeted subunits in all patients.

Supplementary Figure 7.3.3 Incidence of type γ adverse events using different anti-IL-23 agents in all patients.

Supplementary Figure 7.4.1 Incidence of type δ adverse events symptoms using different anti-IL-23 agents in all patients.

Supplementary Figure 7.4.2 Incidence of type δ adverse events using different targeted subunits in all patients.

Supplementary Figure 7.4.3 Incidence of type δ adverse events using different anti-IL-23 agents in all patients.

Supplementary Figure 7.5.1 Incidence of type ***ϵ*** adverse events symptoms using different anti-IL-23 agents in all patients.

Supplementary Figure 7.5.2 Incidence of type ***ϵ*** adverse events using different targeted subunits in all patients.

Supplementary Figure 7.5.3 Incidence of type ***ϵ*** adverse events using different anti-IL-23 agents in all patients.

## Abbreviations

Pso, psoriasis; RA, rheumatoid arthritis; AS, ankylosing spondylitis; SLE, systemic lupus erythematosus; CD, Crohn’s disease; NSAIDs, nonsteroidal anti-inflammatory drugs; SAID, steroid anti-inflammatory drugs; DMARDs, disease-modifying anti-rheumatic drugs; AEs, adverse events; BAs, biological agents; FDA, food and drug administration; TNFR2, tumor necrosis factor receptor 2; IL-23, Interleukin-23; EMEA, European Agency for the Evaluation of Medicinal Products; PRISMA, Preferred Reporting Items for Systematic Reviews and Meta-analyses; RCT, randomized clinical trial; EMBASE, Excerpta Medica data BASE; WOS, Web of Science; CI, confidence interval; OR, the odds ratio.
