# Supplementary figures and images for: Adverse Events Associated With Anti-IL-23 Agents: Clinical Evidence and Possible Mechanisms
Source: Front Immunol. 2021 Jun 11;12:670398. doi: 10.3389/fimmu.2021.670398 (PMC8226270; doi:10.3389/fimmu.2021.670398)

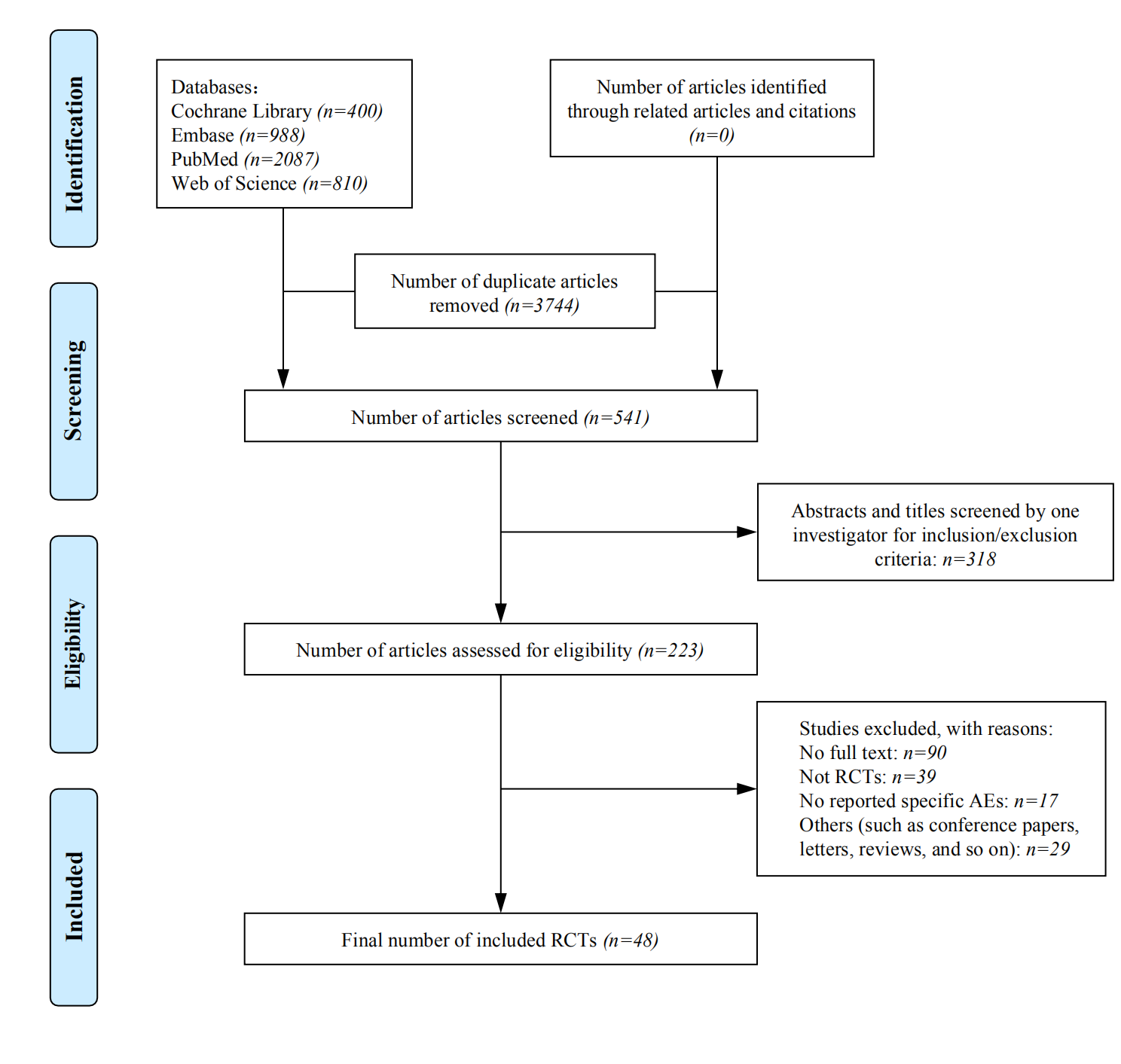

Supplement: Supplementary Figure 1 — Flow diagram of the literature search and selection. [file Image_1.tif]

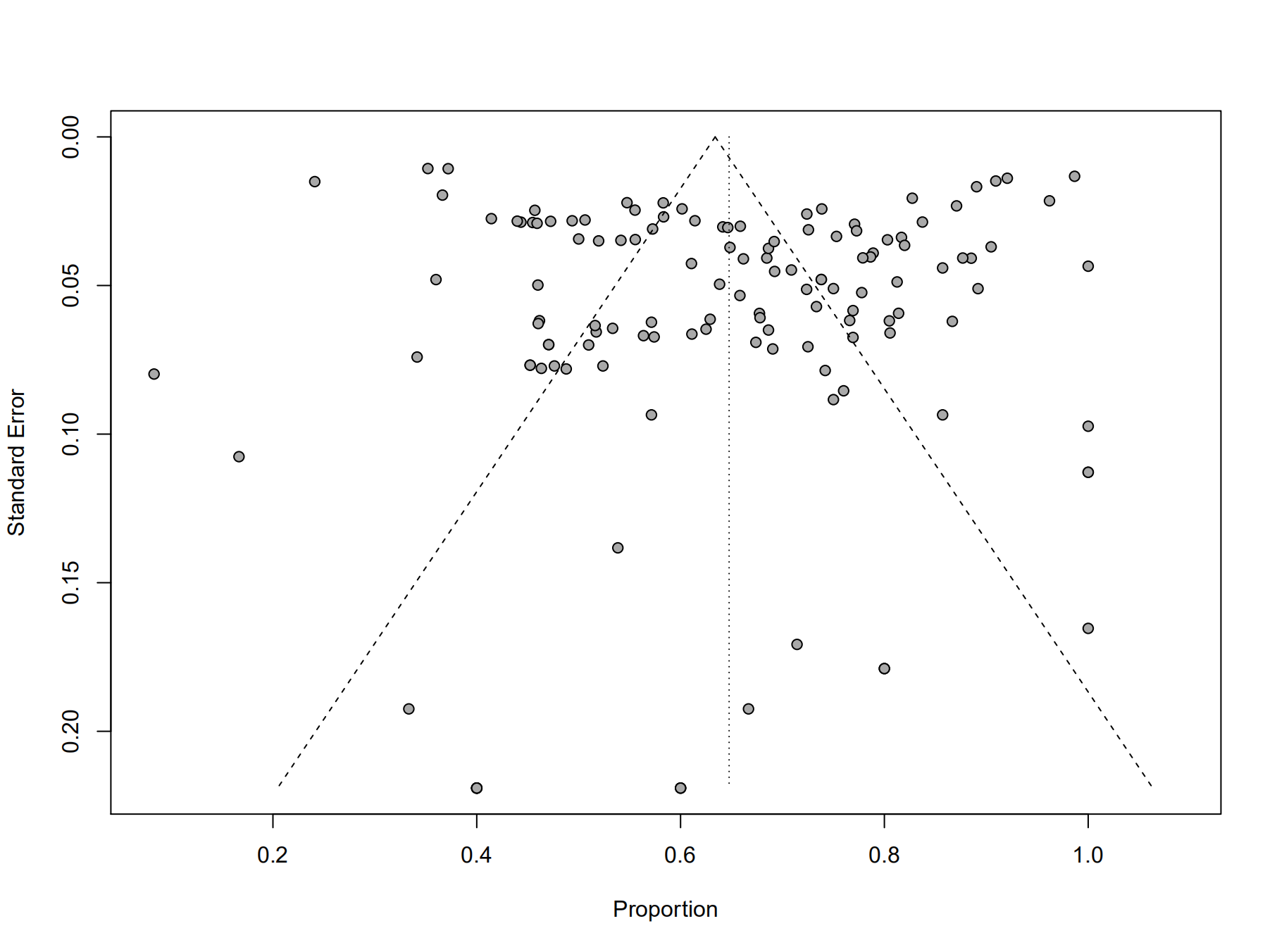

Supplement: Supplementary Figure 2 — Funnel plot [file Image_2.tif]

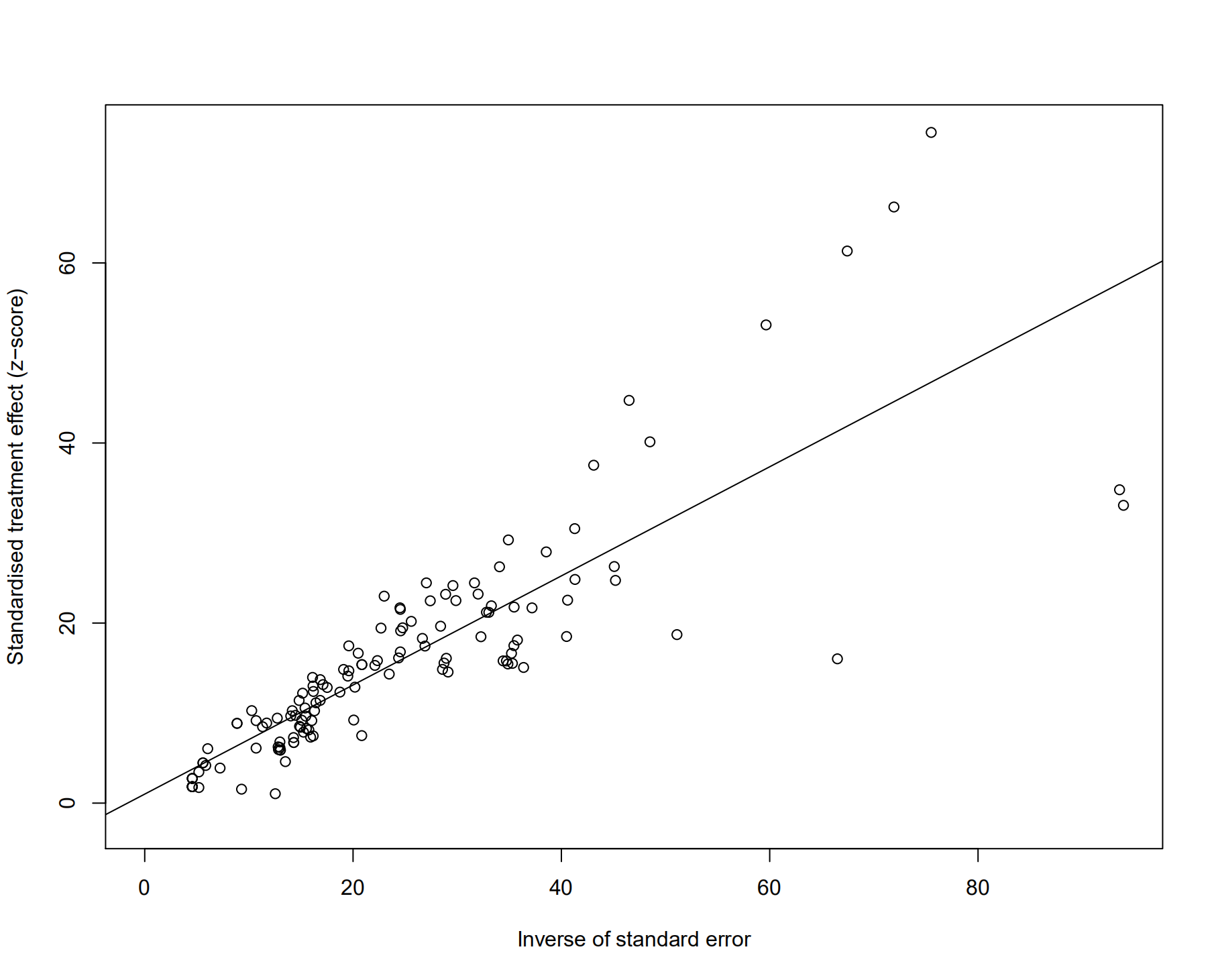

Supplement: Supplementary Figure 3 — Egger’s test [file Image_3.tif]

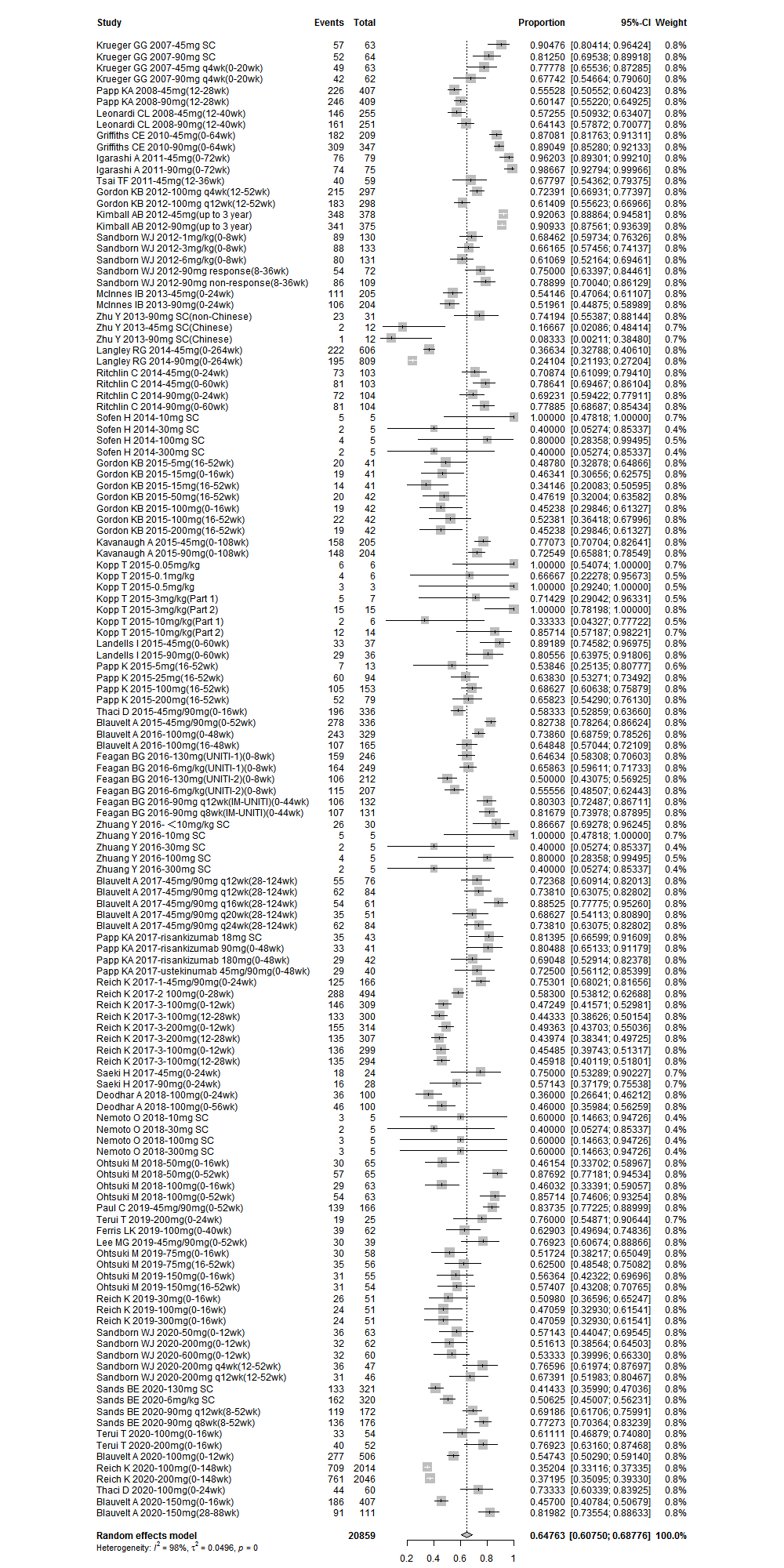

Supplement: Supplementary Figure 4 — Incidence of total adverse events with different anti-IL-23 agents in all patients. [file Image_4.tiff]

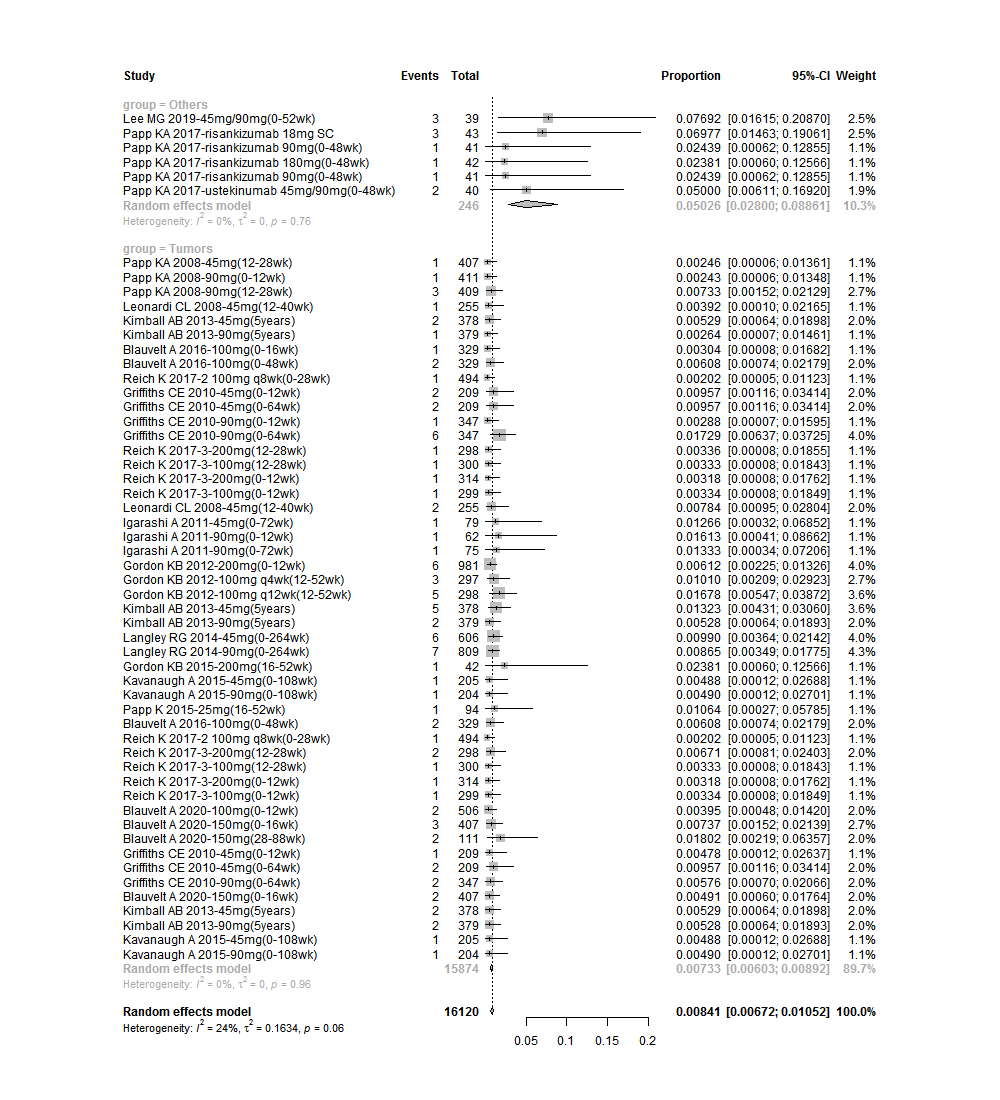

Supplement: Supplementary Figure 5 — Incidence of cancers with different anti-IL-23 agents in all patients. [file Image_5.tiff]

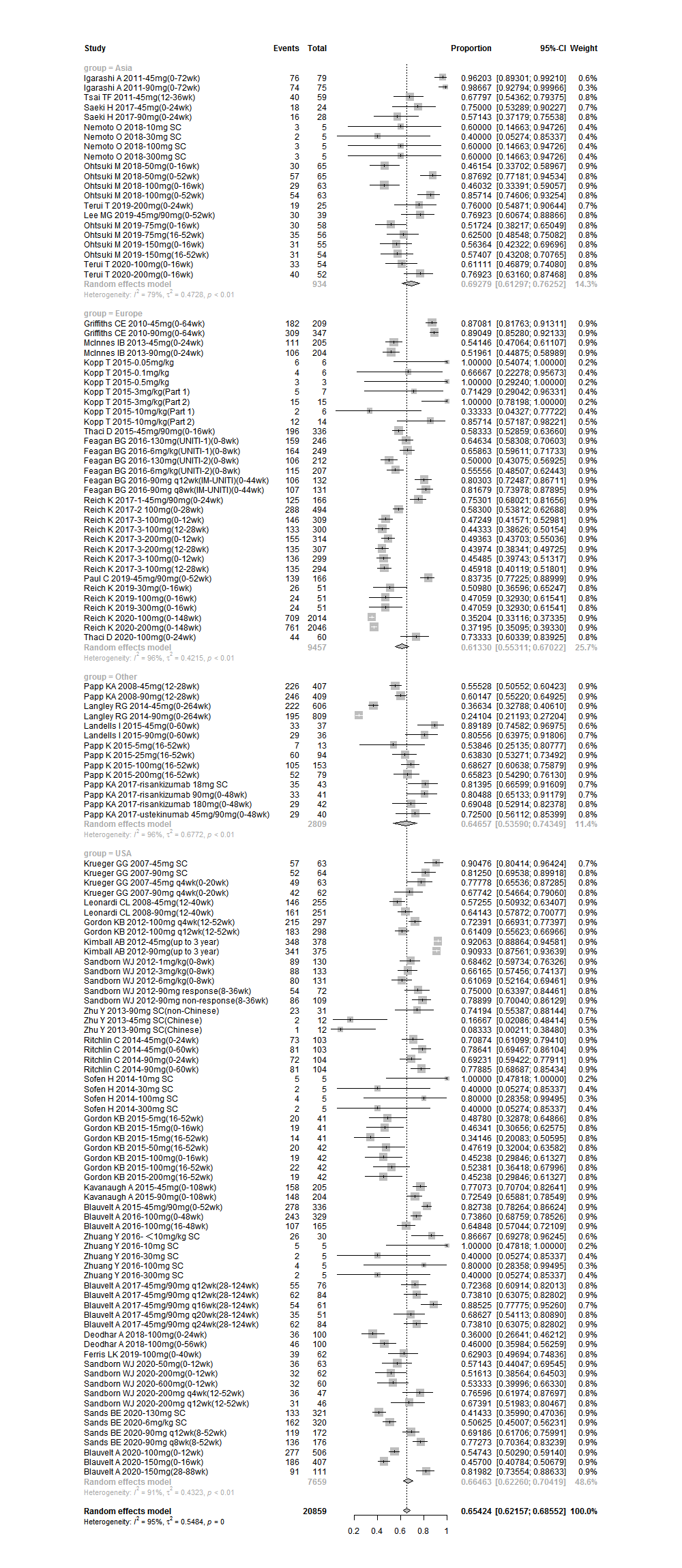

Supplement: Supplementary Figure 6.1 — Incidence of adverse events in different regions [file Image_6.tiff]

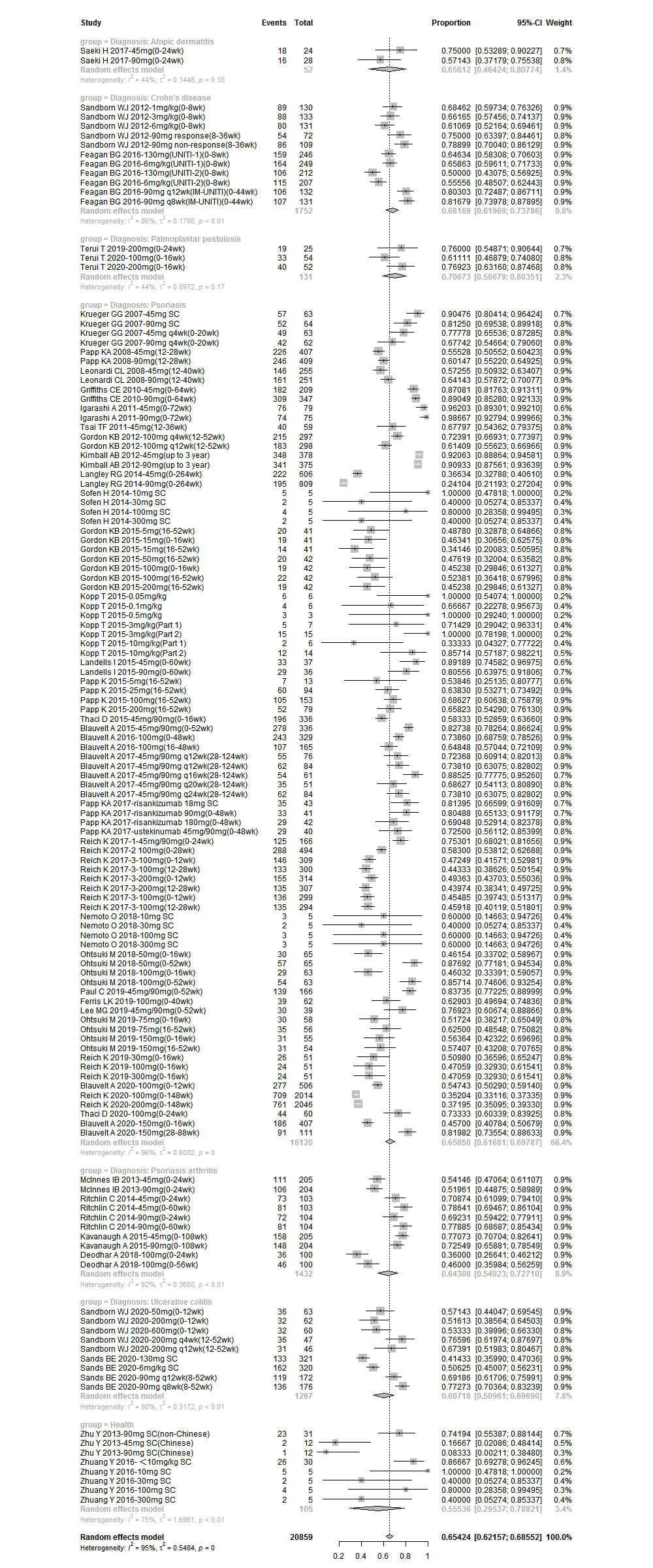

Supplement: Supplementary Figure 6.2 — Incidence of adverse events with different. diagnoses in all patients [file Image_7.tiff]

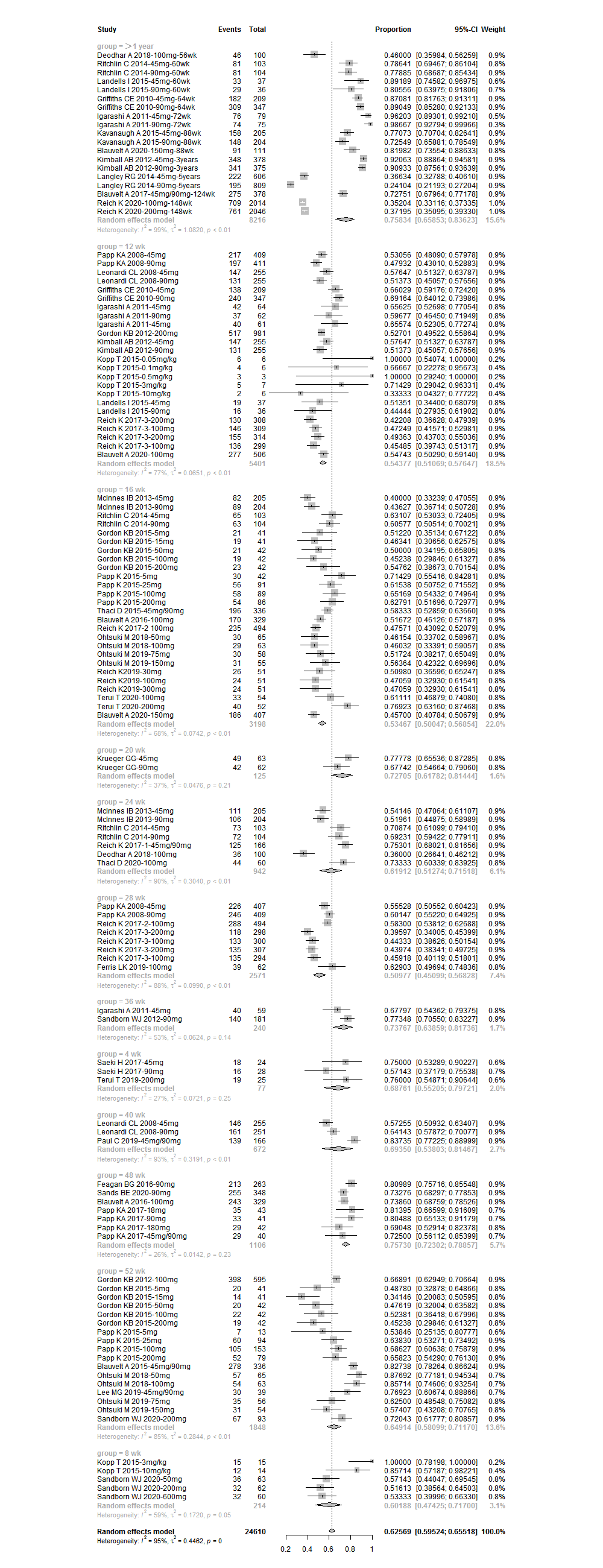

Supplement: Supplementary Figure 6.3 — Incidence of adverse events with different courses of medication in all patients. [file Image_8.tiff]

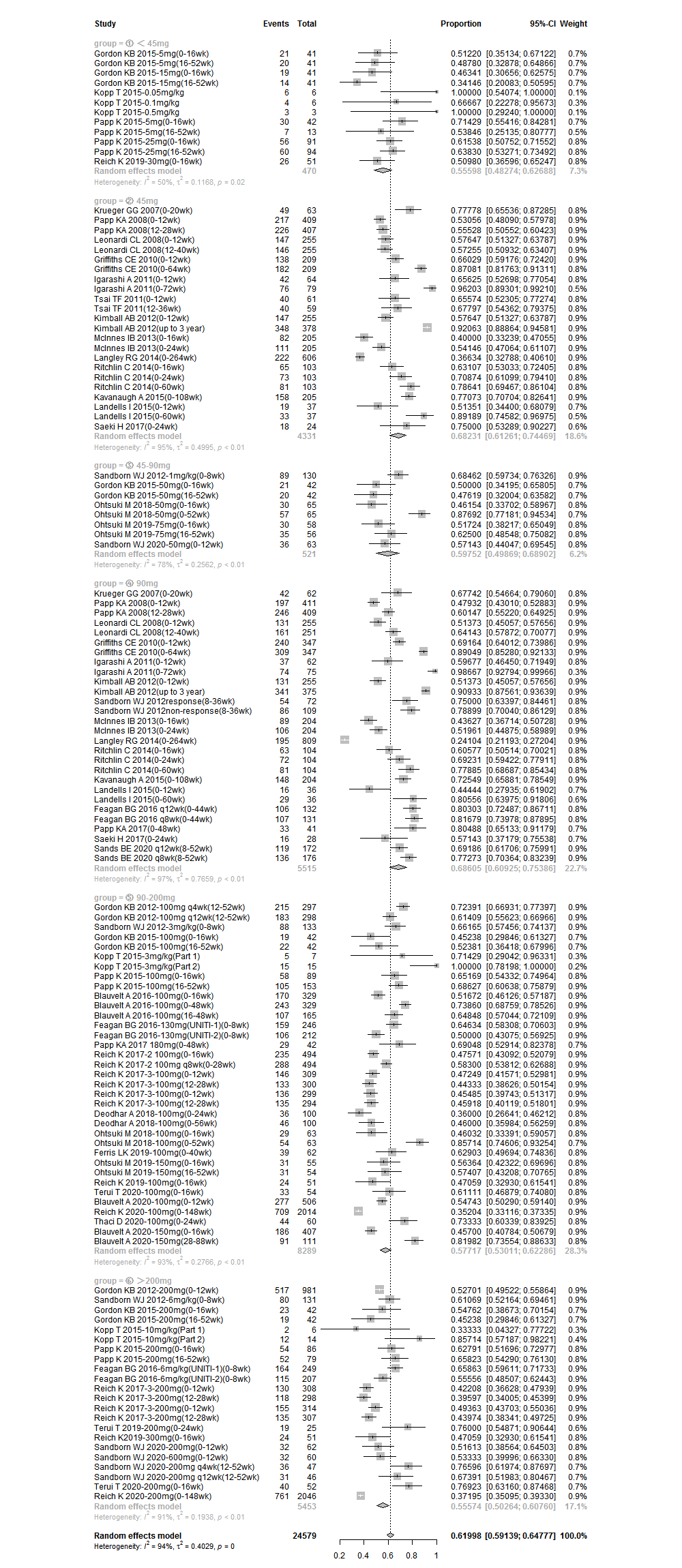

Supplement: Supplementary Figure 6.4.1 — Incidence of adverse events with different therapeutic doses in all patients. [file Image_9.tiff]

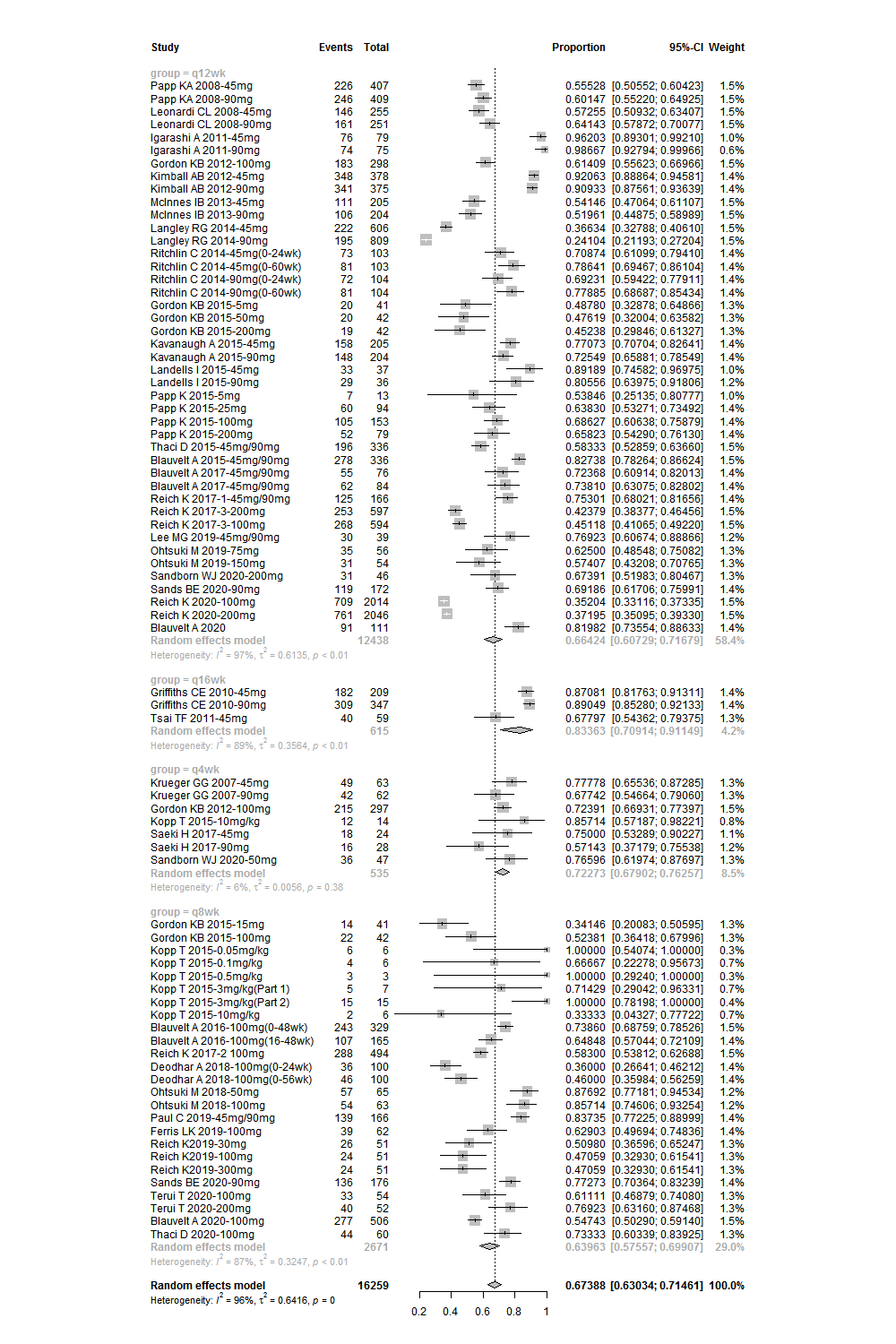

Supplement: Supplementary Figure 6.4.2 — Incidence of adverse events with different frequency of application in all patients. [file Image_10.tiff]

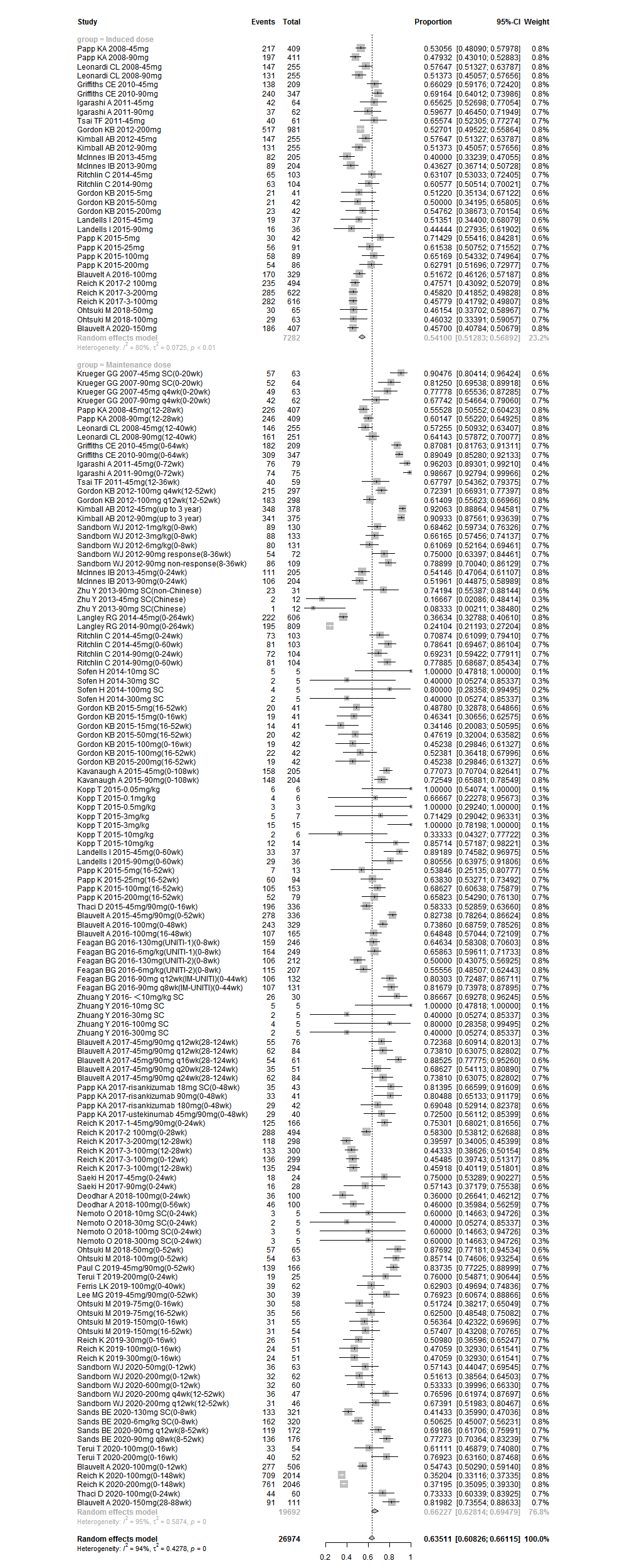

Supplement: Supplementary Figure 6.4.3 — Incidence of adverse events in the induction and maintenance periods in all patients. [file Image_11.tiff]

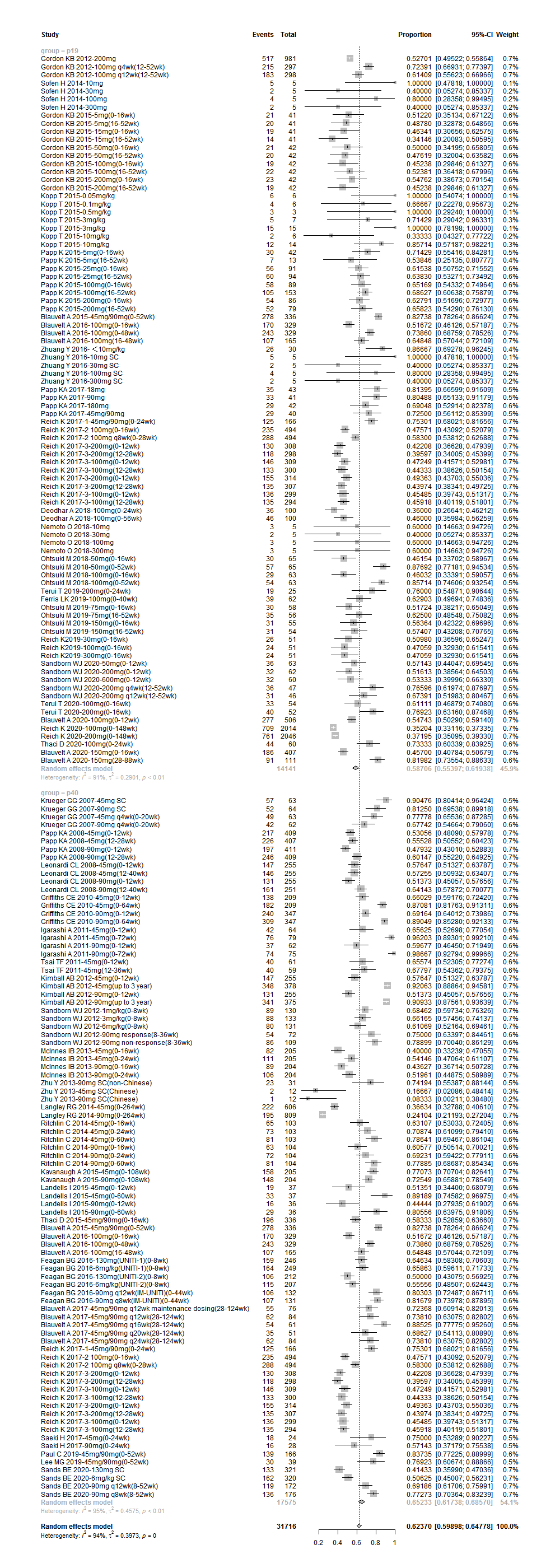

Supplement: Supplementary Figure 6.5.1 — Incidence of adverse events with different targeted subunits in all patients. [file Image_12.tiff]

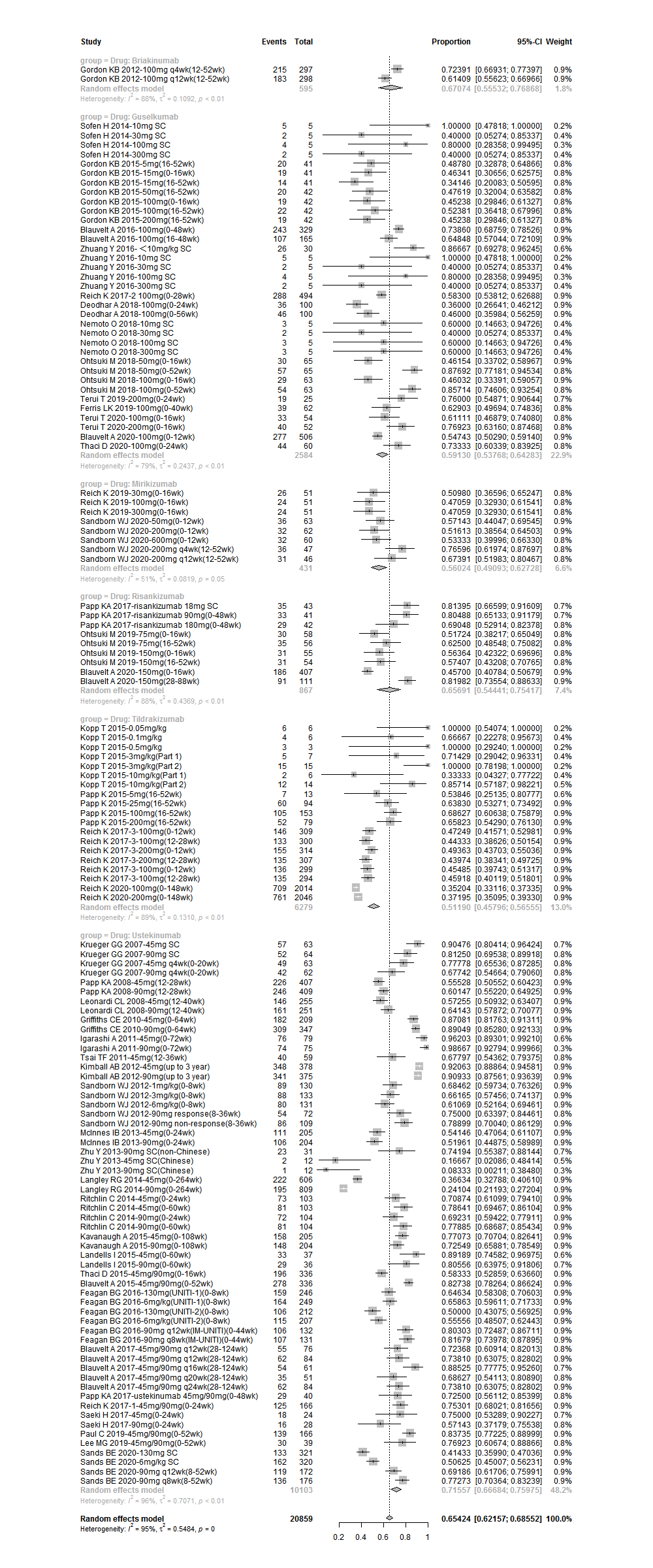

Supplement: Supplementary Figure 6.5.2 — Incidence of adverse events with different anti-IL-23 agents in all patients. [file Image_13.tiff]

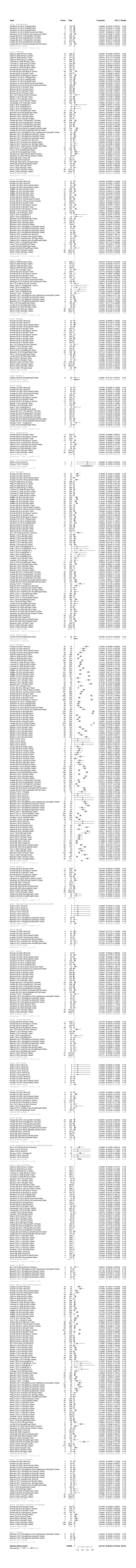

Supplement: Supplementary Figure 7.1.1 — Incidence of type α adverse events symptoms using different anti-IL-23 agents in all patients. [file Image_15.png]
