# Supplementary material for: Adverse Events Associated With Anti-IL-23 Agents: Clinical Evidence and Possible Mechanisms
Source: Front Immunol. 2021 Jun 11;12:670398. doi: 10.3389/fimmu.2021.670398 (PMC8226270; doi:10.3389/fimmu.2021.670398)
Supplement: Supplementary file 16 [file Table_1.doc]

**Table.S1 Classification of adverse events of biological agents**

| **Type** | **Description** | **Performances** |
| --- | --- | --- |
| α | The cytokine release syndrome | Systemic inflammatory response: fever, fatigue, arthritis, headache, myalgia, gastrointestinal symptoms (nausea, vomiting, diarrhea), pulmonary edema, encephalopathy, etc.. |
| β | Hypersensitivity reactions | Immediate (IgE): urticaria, allergic asthma, allergic rhinitis, eosinophilia, granulocyte dysfunction, etc..  Delayed (IgG, T cell): serum sickness, vasculitis, nephritis, etc.. |
| γ | Immune/cytokine imbalance syndromes;  (chemical) reactions | Thrombocytopenia, hemolytic anemia, IgA nephropathy, herpetic dermatitis, systemic lupus erythematosus, vasculitis, thyroid disease, pernicious anemia, psoriasis, vitiligo, interstitial lung disease, multiple sclerosis, etc.. |
| δ | Cross-reactivity; (delayed) reactions | Malignant tumor, acne, etc.. |
| ***ε*** | Non-imunological side-effects | Heart failure, psychiatric disorders, paraesthesia, hearing loss, etc.. |
