# Supplementary material for: Adverse Events Associated With Anti-IL-23 Agents: Clinical Evidence and Possible Mechanisms
Source: Front Immunol. 2021 Jun 11;12:670398. doi: 10.3389/fimmu.2021.670398 (PMC8226270; doi:10.3389/fimmu.2021.670398)
Supplement: Supplementary file 17 [file Table_2.doc]

**Table.S2 The Cochrane Collaboration’s tool for assessing risk of bias of RCTs.**

|  | Random sequence generation | Allocation concealment | Blinding of participants and personnel | Blinding of outcome assessment | Incomplete outcome data | Selective outcome reporting | Other source of bias |
| --- | --- | --- | --- | --- | --- | --- | --- |
| Krueger GG 2007 | + | + | + | + | + | + | ? |
| Papp KA 2008 | + | + | + | + | + | + | ? |
| Leonardi CL 2008 | + | + | + | + | + | + | ? |
| Griffiths CE 2010 | + | ? | + | + | + | + | ? |
| Igarashi A 2011 | + | + | + | + | + | + | ? |
| Tsai TF 2011 | + | ? | + | + | + | + | ? |
| Gordon KB 2012 | + | + | + | + | + | + | ? |
| Kimball AB 2012 | + | ? | + | + | + | + | ? |
| Sandborn WJ 2012 | + | + | + | + | + | + | ? |
| Kimball AB 2013 | ? | ? | - | - | + | + | ? |
| McInnes IB 2013 | + | + | + | + | + | + | ? |
| Zhu Y 2013 | ? | - | - | - | + | + | ? |
| Langley RG 2014 | + | + | + | - | + | + | ? |
| Ritchlin C 2014 | + | + | - | - | + | + | ? |
| Sofen H 2014 | ? | ? | + | + | + | + | ? |
| Gordon KB 2015 | ? | ? | + | + | + | + | ? |
| Kavanaugh A 2015 | + | ? | + | + | + | + | ? |
| Kopp T 2015 | ? | ? | ? | ? | + | + | ? |
| Landells I 2015 | + | ? | + | + | + | + | ? |
| Papp K 2015 | + | + | + | + | + | + | ? |
| Thaçi D 2015 | + | ? | + | + | + | + | ? |
| Blauvelt A 2015 | + | ? | + | + | + | + | ? |
| Blauvelt A 2016 | + | + | + | + | + | + | ? |
| Feagan BG 2016 | + | + | + | + | + | + | ? |
| Zhuang Y 2016 | + | + | + | + | + | + | ? |
| Blauvelt A 2017 | ? | ? | + | + | + | + | ? |
| Papp KA 2017 | ? | ? | + | + | + | + | ? |
| Reich K 2017-1 | + | + | + | + | + | + | ? |
| Reich K 2017-2 | + | + | + | + | + | + | ? |
| Reich K 2017-3 | + | + | + | + | + | + | ? |
| Saeki H 2017 | + | ? | + | + | + | + | ? |
| Deodhar A 2018 | + | + | + | + | + | + | ? |
| Nemoto O 2018 | + | + | + | + | + | + | ? |
| Ohtsuki M 2018 | + | + | + | + | + | + | ? |
| Paul C 2019 | - | ? | + | + | + | + | ? |
| Terui T 2019 | + | + | + | + | + | + | ? |
| Ferris LK 2019 | + | + | + | + | + | + | ? |
| Lee MG 2019 | - | ? | ? | ? | + | + | ? |
| Ohtsuki M 2019 | + | ? | + | + | + | + | ? |
| Reich K2019 | + | + | + | + | + | + | ? |
| Sandborn WJ 2020 | + | + | + | + | + | + | ? |
| Sands BE 2020 | + | + | + | + | + | + | ? |
| Terui T 2020 | + | ? | + | + | + | + | ? |
| Blauvelt A 2020 | + | + | + | + | + | + | ? |
| Gelfand JM 2020 | ? | ? | ? | ? | + | + | ? |
| Reich K 2020 | + | + | + | + | + | + | ? |
| Thaçi D 2020 | + | + | + | + | + | + | ? |
| Blauvelt A 2020 | - | + | + | + | + | + | ? |

The dark green cells (+) indicate a low risk of bias. The red cells (-) indicate a high risk of bias. The yellow cells (?) indicate an uncertain risk of bias. n/a, not applicable
