# Supplementary material for: Adverse Events Associated With Anti-IL-23 Agents: Clinical Evidence and Possible Mechanisms
Source: Front Immunol. 2021 Jun 11;12:670398. doi: 10.3389/fimmu.2021.670398 (PMC8226270; doi:10.3389/fimmu.2021.670398)
Supplement: Supplementary file 18 [file Table_3.docx]

**Table.S3** Estimates of adverse events in 48 included studies

| **Trials** | **Any Grade Adverse Events** | | | | |
| --- | --- | --- | --- | --- | --- |
|  | **Studies** | **Patients** | **Incidence** | ***I^2^*** | ***P* Value** |
| **1. Regions** | | | | | |
| **U.S.A** | 20 | 7,659 | 66.46 [62.26, 70.42] | 91 | ＜0.01 |
| **Europe** | 11 | 9,457 | 61.33 [55.31, 67.02] | 96 | ＜0.01 |
| **Asia** | 9 | 934 | 69.28 [61.30, 76.25] | 79 | ＜0.01 |
| **Other** | 5 | 2,809 | 64.66 [53.59, 74.35] | 96 | ＜0.01 |
| **2.** **Diagnosis** | | | | | |
| **Atopic dermatitis** | 1 | 52 | 65.61 [46.42, 80.78] | 44 | 0.18 |
| **Crohn’s disease** | 2 | 1,752 | 68.17 [61.97, 73.79] | 86 | ＜0.01 |
| **Palmoplantar pustulosis** | 2 | 131 | 70.67 [58.68, 80.35] | 44 | 0.17 |
| **Psoriasis** | 33 | 16,120 | 65.85 [61.68, 69.79] | 96 | 0 |
| **Psoriasis arthritis** | 4 | 1,432 | 64.31 [54.92, 72.71] | 92 | ＜0.01 |
| **Ulcerative colitis** | 2 | 1,267 | 60.72 [50.96, 69.69] | 90 | ＜0.01 |
| **Health** | 1 | 105 | 53.54 [29.54, 78.82] | 75 | ＜0.01 |
| **3. Courses of Medication** | | | | | |
| **4 weeks** | 2 | 77 | 68.76 [55.21, 79.72] | 27 | 0.25 |
| **8 weeks** | 2 | 214 | 60.19 [47.43, 71.70] | 59 | 0.05 |
| **12 weeks** | 10 | 5,401 | 54.38 [51.07, 57.65] | 77 | ＜0.01 |
| **16 weeks** | 12 | 3,198 | 53.47 [50.05, 56.85] | 68 | ＜0.01 |
| **20 weeks** | 1 | 125 | 72.71 [61.78, 81.44] | 37 | 0.21 |
| **24 weeks** | 5 | 942 | 61.91 [51.27, 71.52] | 90 | ＜0.01 |
| **28 weeks** | 4 | 2,571 | 50.98 [45.10, 56.83] | 88 | ＜0.01 |
| **36 weeks** | 2 | 240 | 73.77 [63.86, 81.74] | 53 | 0.14 |
| **40 weeks** | 2 | 672 | 69.35 [53.80, 81.47] | 93 | ＜0.01 |
| **48 weeks** | 4 | 1,106 | 75.73 [72.30, 78.86] | 26 | 0.23 |
| **52 weeks** | 8 | 1,848 | 64.91 [58.10, 71.17] | 85 | ＜0.01 |
| **＞ 1 year** | 11 | 8,216 | 75.83 [65.85, 83.62] | 99 | ＜0.01 |
| **4. Dose Adjustment** |  |  |  |  |  |
| **Dose** |  |  |  |  |  |
| ＜ 45 mg | 3 | 470 | 55.60 [48.27, 62.69] | 50 | 0.02 |
| 45 mg | 13 | 4,331 | 68.23 [61.26, 74.70] | 95 | ＜0.01 |
| 90 mg | 16 | 5,515 | 68.61 [60.93, 75.39] | 97 | ＜0.01 |
| 90-200 mg | 20 | 8,289 | 57.72 [53.01, 62.29] | 93 | ＜0.01 |
| ＞ 200 mg | 12 | 5,453 | 55.58 [50.26, 60.76] | 91 | ＜0.01 |
| **Frequency of application** |  |  |  |  |  |
| q4wk | 5 | 535 | 72.27 [67.90, 76.26] | 6 | 0.38 |
| q8wk | 13 | 2,671 | 63.96 [57.56, 69.91] | 87 | ＜0.01 |
| q12wk | 23 | 12,438 | 66.42 [60.73, 71.68] | 97 | ＜0.01 |
| q16wk | 2 | 615 | 83.36 [70.91, 91.15] | 89 | ＜0.01 |
| **Treatment period** |  |  |  |  |  |
| Induced period | 17 | 7,282 | 54.10 [51.28, 56.89] | 80 | ＜0.01 |
| Maintenance period | 43 | 19,692 | 66.23 [62.81, 69.48] | 95 | 0 |
| **5. Drugs** |  |  |  |  |  |
| **p19** | **22** | **14,141** | **58.71 [55.40, 61.94]** | **91** | **＜0.01** |
| Guselkumab | 13 | 2,584 | 59.13 [53.77, 64.28] | 79 | ＜0.01 |
| Mirikizumab | 2 | 431 | 56.02 [49.09, 62.73] | 51 | 0.05 |
| Risankizumab | 3 | 867 | 65.69 [54.44, 75.42] | 88 | ＜0.01 |
| Tildrakizumab | 4 | 6,279 | 51.19 [45.80, 56.56] | 89 | ＜0.01 |
| **p40** | **27** | **17,575** | **65.23 [61.74, 68.57]** | **95** | **＜0.01** |
| Ustekinumab | 26 | 10,103 | 71.56 [66.68, 75.98] | 96 | ＜0.01 |
| Briakinumab | 1 | 595 | 67.07 [55.53, 76.87] | 88 | ＜0.01 |
| **6. Types of AEs** | | | | | |
| **Type α** | **42** | **157,159** | **7.14 [6.41, 7.93]** | **97** | **0** |
| p19 | 21 | 63,554 | 9.17 [7.65, 10.96] | 96 | 0 |
| p40 | 22 | 93,605 | 6.48 [5.72, 7.34] | 96 | 0 |
| **Type β** | **40** | **62,810** | **6.63 [5.86, 7.49]** | **90** | **＜0.01** |
| p19 | 20 | 32,858 | 6.87 [5.47, 8.60] | 90 | ＜0.01 |
| p40 | 21 | 29,952 | 6.65 [5.79, 7.62] | 90 | ＜0.01 |
| **Type γ** | **31** | **25,698** | **3.86 [3.26, 4.57]** | **80** | **＜0.01** |
| p19 | 15 | 8,279 | 3.77 [2.80, 5.07] | 65 | ＜0.01 |
| p40 | 17 | 17,419 | 3.90 [3.21, 4.74] | 81 | ＜0.01 |
| **Type δ** | **15** | **16,120** | **0.84 [0.67, 1.05]** | **24** | **0.06** |
| p19 | 7 | 5,581 | 1.01 [0.70, 1.46] | 32 | 0.09 |
| p40 | 9 | 10,539 | 0.75 [0.57, 10.00] | 19 | 0.161 |
| **Type Σ** | **7** | **816** | **13.30 [6.23, 26.17]** | **77** | **＜0.01** |
| p19 | 6 | 619 | 15.17 [6.72, 30.76] | 75 | ＜0.01 |
| p40 | 2 | 197 | 4.85 [0.10, 72.83] | 93 | ＜0.01 |
| **6.1 Type α** | | | | | |
| Abdominal pain | 3 | 1,621 | 5.37 [4.10, 6.99] | 36 | 0.12 |
| Arthralgia | 21 | 14,753 | 5.02 [4.22, 5.95] | 78 | ＜0.01 |
| Backache | 14 | 10,490 | 4.52 [3.53, 5.77] | 81 | ＜0.01 |
| Cough | 12 | 7,874 | 3.92 [2.87, 5.33] | 76 | ＜0.01 |
| Diarrhea | 13 | 8,176 | 3.72 [2.94, 4.70] | 64 | ＜0.01 |
| Dizziness | 1 | 22 | 9.09 [2.28, 3.00] | NA | NA |
| Fatigue | 8 | 3,319 | 3.67 [2.98, 4.53] | 18 | 0.23 |
| Gastroenteritis | 4 | 5,529 | 3.59 [2.05, 6.23] | 90 | ＜0.01 |
| Gastrointestinal disorders | 2 | 10 | 60.00 [29.74, 84.17] | 0 | 1 |
| Headache | 26 | 18,928 | 7.12 [6.16, 8.23] | 82 | ＜0.01 |
| Herpes zoster | 1 | 39 | 7.69 [2.50, 21.30] | NA | NA |
| Infections | 32 | 19,282 | 36.35 [32.41, 40.48] | 96 | 0 |
| Influenza | 6 | 6,615 | 3.99 [2.64, 5.97] | 86 | ＜0.01 |
| Musculoskeletal and connective tissue  disorders | 3 | 249 | 8.57 [4.56, 15.55] | 26 | 0.21 |
| Myalgia | 2 | 375 | 5.58 [3.58, 8.59] | 0 | 0.81 |
| Nausea | 9 | 7,599 | 3.76 [2.82, 4.99] | 73 | ＜0.01 |
| Oropharyngeal pain | 8 | 5,982 | 3.54 [2.35, 5.28] | 76 | ＜0.01 |
| Other inguries | 3 | 282 | 7.65 [4.06, 13.96] | 27 | 0.19 |
| Other pains | 6 | 1,257 | 5.25 [3.53, 7.76] | 47 | 0.05 |
| Pyrexia | 2 | 2,166 | 4.15 [2.88, 6.00] | 64 | ＜0.01 |
| Respiratory, thoracic and mediastinal  disorders | 3 | 81 | 26.13 [17.68, 36.81] | 0 | 0.99 |
| Serious infections | 15 | 11,067 | 1.23 [0.95, 1.60] | 45 | ＜0.01 |
| Toothache | 2 | 387 | 3.77 [1.98, 7.07] | 21 | 0.28 |
| Upper respiratory tract infection | 29 | 20,014 | 8.53 [7.18, 10.11] | 91 | ＜0.01 |
| Urinary tract infection | 7 | 5,069 | 2.72 [2.07, 3.56] | 23 | 0.18 |
| Viral upper respiratory tract infection | 4 | 5,538 | 6.73 [5.22, 8.66] | 71 | ＜0.01 |
| Vomiting | 1 | 244 | 4.04 [2.11, 7.58] | 0 | 0.42 |
| **6.2 Type β** | | | | | |
| Anaphylaxis | 44 | 865 | 5.00 [1.77, 13.34] | 88 | ＜0.01 |
| Bronchitis | 7 | 6,176 | 3.96 [2.70, 5.76] | 80 | ＜0.01 |
| Contact dermatitis | 2 | 1,033 | 3.84 [2.12, 6.85] | 59 | 0.06 |
| Eczema | 4 | 233 | 6.72 [4.01, 11.05] | 0 | 0.63 |
| Eosinophilia | 1 | 120 | 4.26 [1.78, 9.84] | 0 | 0.62 |
| Erythema | 10 | 8,807 | 2.51 [1.66, 3.77] | 81 | ＜0.01 |
| Infusion reaction | 1 | 394 | 4.37 [2.73, 6.92] | 0 | 0.78 |
| Injection-site reaction | 21 | 8,819 | 4.06 [2.59, 6.32] | 88 | ＜0.01 |
| Ieukopenia | 3 | 381 | 4.85 [2.23, 10.25] | 53 | 0.09 |
| Nasopharyngitis | 34 | 23,123 | 12.21 [10.71, 13.89] | 91 | ＜0.01 |
| Pharyngitis | 3 | 314 | 5.39 [3.21, 8.90] | 0 | 0.46 |
| Pruritus | 9 | 5,538 | 4.40 [2.86, 6.71] | 79 | ＜0.01 |
| Rhinitis | 6 | 781 | 5.82 [4.31, 7.82] | 0 | 0.71 |
| Sinusitis | 5 | 6,056 | 4.57 [2.93, 7.05] | 88 | ＜0.01 |
| Urticaria | 3 | 170 | 6.00 [3.14, 11.13] | 0 | 0.61 |
| **6.3 Type γ** | | | | | |
| Anemia | 3 | 1,294 | 3.41 [2.53, 4.61] | 0 | 0.56 |
| Cardiovascular events | 14 | 7,925 | 0.76 [049, 1.18] | 49 | ＜0.01 |
| Crohn’s disease | 3 | 1,918 | 5.96 [3.88, 9.07] | 80 | ＜0.01 |
| Elevated transaminases | 4 | 615 | 7.43 [3.61, 14.67] | 82 | ＜0.01 |
| Epistaxis | 1 | 12 | 8.33 [1.16, 41.32] | NA | NA |
| Hepatic events | 2 | 672 | 1.82 [1.04, 3.18] | 0 | 0.49 |
| Hepatic steatosis | 1 | 200 | 2.28 [0.86, 5.91] | 0 | 0.34 |
| Hyperglycemia | 1 | 120 | 6.84 [3.46, 13.09] | 0 | 0.5 |
| Hypertension | 9 | 6,628 | 5.50 [4.09, 7.34] | 78 | ＜0.01 |
| Increased blood creatine phosphokinase | 3 | 372 | 9.03 [4.90, 16.05] | 57 | 0.02 |
| Increased blood triglycerides | 1 | 218 | 15.80 [9.83, 24.42] | 52 | 0.12 |
| Psoriasis | 11 | 3,677 | 3.92 [3.05, 5.02] | 45 | ＜0.01 |
| Purpura | 1 | 252 | 5.79 [3.39, 9.72] | 0 | 0.5 |
| Renal and urinary disorders | 2 | 10 | 20.00 [5.04, 54.07] | 0 | 1 |
| Serious tuberculosis | 1 | 518 | 0.75 [0.11, 5.09] | 63 | 0.1 |
| Ulcerative colitis | 2 | 1,267 | 5.28 [3.05, 9.00] | 76 | ＜0.01 |
| **6.4 Type δ** | | | | | |
| Acne | 1 | 39 | 7.70 [2.50, 21.30] | NA | NA |
| B cell lymphoma | 1 | 409 | 0.49 [0.12, 1.93] | 0 | 1 |
| Cutaneous cancer | 2 | 1,482 | 0.46 [0.21, 1.02] | 0 | 0.69 |
| Dental caries | 1 | 81 | 3.93 [1.27, 11.49] | 0 | 0.55 |
| Folliculitis | 1 | 126 | 4.53 [1.89, 10.46] | 0 | 0.49 |
| Malignancies | 15 | 9,751 | 0.80 [0.63, 1.01] | 0 | 0.97 |
| NMSC | 5 | 4,232 | 0.66 [0.44, 1.01] | 0 | 0.47 |
| **6.5 Type Σ** | | | | | |
| Anxiety | 1 | 22 | 9.09 [2.28, 29.96] | NA | NA |
| Depression | 1 | 672 | 0.75 [0.31, 1.79] | 0 | ＜0.01 |
| Insomnia | 1 | 6 | 33.33 [8.39, 73.19] | NA | NA |
| Metabolism and nutrition disorders | 2 | 10 | 20.00[5.04, 54.07] | 0 | 1 |
| Nervous system disorders | 3 | 81 | 27.49 [18.80, 38.30] | 0 | 0.95 |
| Paraesthesia | 1 | 15 | 13.33 [3.36, 40.54] | NA | NA |
| Psychiatric disorders | 2 | 10 | 20.00 [5.04, 54.07] | 0 | 1 |
